# A Systems Approach Reveals Regulatory Circuitry for *Arabidopsis* Trichome Initiation by the GL3 and GL1 Selectors

**DOI:** 10.1371/journal.pgen.1000396

**Published:** 2009-02-27

**Authors:** Kengo Morohashi, Erich Grotewold

**Affiliations:** 1 Department of Plant, Cellular, and Molecular Biology, Ohio State University, Columbus, Ohio, United States of America; 2 Plant Biotechnology Center, Ohio State University, Columbus, Ohio, United States of America; 3 Mathematical Bioscience Institute, Ohio State University, Columbus, Ohio, United States of America; The Salk Institute for Biological Studies, United States of America

## Abstract

Position-dependent cell fate determination and pattern formation are unique aspects of the development of plant structures. The establishment of single-celled leaf hairs (trichomes) from pluripotent epidermal (protodermal) cells in *Arabidopsis* provides a powerful system to determine the gene regulatory networks involved in cell fate determination. To obtain a holistic view of the regulatory events associated with the differentiation of *Arabidopsis* epidermal cells into trichomes, we combined expression and genome-wide location analyses (ChIP-chip) on the trichome developmental selectors *GLABRA3* (*GL3*) and *GLABRA1* (*GL1*), encoding basic helix-loop-helix (bHLH) and MYB transcription factors, respectively. Meta-analysis was used to integrate genome-wide expression results contrasting wild type and *gl3* or *gl1* mutants with changes in gene expression over time using inducible versions of GL3 and GL1. This resulted in the identification of a minimal set of genes associated with the differentiation of epidermal cells into trichomes. ChIP-chip experiments, complemented by the targeted examination of factors known to participate in trichome initiation or patterning, identified about 20 novel GL3/GL1 direct targets. In addition to genes involved in the control of gene expression, such as the transcription factors *SCL8* and *MYC1*, we identified *SIM* (*SIAMESE*), encoding a cyclin-dependent kinase inhibitor, and *RBR1* (*RETINOBLASTOMA RELATED1*), corresponding to a negative regulator of the cell cycle transcription factor E2F, as GL3/GL1 immediate targets, directly implicating these trichome regulators in the control of the endocycle. The expression of many of the identified GL3/GL1 direct targets was specific to very early stages of trichome initiation, suggesting that they participate in some of the earliest known processes associated with protodermal cell differentiation. By combining this knowledge with the analysis of genes associated with trichome formation, our results reveal the architecture of the top tiers of the hierarchical structure of the regulatory network involved in epidermal cell differentiation and trichome formation.

## Author Summary

The establishment of single-celled leaf hairs (trichomes) from pluripotent epidermal (protodermal) cells provides a powerful system to determine the gene regulatory networks involved in plant cell fate determination. Two transcription factors—GL1 and GL3—have been associated with the initiation of trichome formation; yet only a handful of GL1-GL3–regulated genes have previously been characterized. In this study, we combined expression analyses performed in a number of different genotypes to identify a minimal set of about 500 genes associated with trichome formation. We also used ChIP-chip to identify a set of about 20 genes that are immediate targets of GL3 and GL1. Many more genes are targeted by GL1 or by GL3, likely in cooperation with other bHLH of MYB partners, but not by both GL1 and GL3. As predicted for genes involved in the initiation of epidermal cell fate determination, several of the GL3/GL1 direct targets are expressed early during trichome formation, including the transcription factors MYC1 (bHLH), SCL8 (GRAS), and genes involved in the regulation of the endocycle (*SIM* and *RBR1*). Co-expression analyses permitted us to identify sets of target genes likely downstream of the GL3/GL1 regulated transcription factors, providing the first steps towards building the regulatory network associated with the differentiation of protodermal cells into trichomes.

## Introduction

Position-dependent cell fate determination and pattern formation are unique aspects of the development of plant structures. The establishment of single-celled leaf hairs (trichomes) from pluripotent epidermal (protodermal) cells provides a powerful system to determine the genetic networks and positional cues involved in cell fate determination [1]–[4]. In the *Arabidopsis* leaf, trichomes constitute the first differentiated cell type. While the number is variable between different leaves and ecotypes, trichomes represent 1–2% of the roughly 1.2×10^4^ cells that constitute the *Arabidopsis* leaf adaxial epidermis. In a developing *Arabidopsis* leaf, mature trichomes first appear at the tip of the young leaf resulting in a progression of younger trichomes towards the base of the leaf. Mature trichomes are characterized by the presence of a stalk with 2–4 branches and an average DNA content of 32 C [5]. Because of the ease to score mutants, ∼70 genes involved in various aspects of trichome initiation, spacing, size and morphology have been identified [3] (Table S1).

Trichome initiation is regulated by the combinatorial action of the R2R3-MYB GLABRA1 (GL1) together with the bHLH GLABRA3 (GL3) or ENHANCER OF GLABRA3 (EGL3) transcription factors [2], [6]–[11]. While *gl1* mutants are mostly glabrous, mutations in *gl3* have a modest effect, primarily affecting branching, DNA endoreduplication and trichoblast size [5],[8]. In contrast, *egl3* plants have no obvious trichome defect, but *gl3 egl3* double mutants are glabrous [7]. Thus, GL3 and EGL3 have partially redundant functions, yet they display distinct expression patterns during leaf development. Maximum *GL3* and *EGL3* expression is observed in leaf primorida. In mature leaves, *GL3* expression persists in trichomes, while *EGL3* expressed at low levels in both pavement cells and trichomes [12]. Highlighting the central role of GL3 in the selection of protodermal cells to the trichome pathway, four hours of induction of a post-translationally regulated version of GL3 (GL3-GR, where GR corresponds to the ligand-binding domain of the glucocorticoid receptor) are sufficient to trigger, in a discrete region of young leaves, the initiation of the trichome differentiation pathway [13]. Within this timeframe, GL3, in cooperation with GL1, binds to and activates expression of *GLABRA2* (*GL2*) [13], encoding a homeo-domain Zip (HD-Zip) transcription factor [14], as well as *TRANSPARENT TESTA GLABRA2* (TTG2) [12], which encodes a WRKY regulator [15]. Within four hours, GL3 also activates the expression of a subset of partially redundant single-repeat R3 MYB proteins, including CAPRICE (CPC) [16] and ENHANCER OF TRY and CPC1 (ETC1). Similarly as TRIPTYCHON (TRY) [5] and ENHANCER OF TRY and CPC2 (ETC2) [17], CPC and ETC1 play central roles in lateral inhibition, by targeting specific components of the MYB/bHLH/TTG1 regulatory complex, making it non-functional [18]. However, only CPC has so far been shown to move to adjacent cells in the leaf epidermis [12]. *TRY* is also a GL3 direct target, but in contrast to *CPC* and *ETC1*, GL3 binds to the *TRY* promoter at later stages during trichome development and independently of GL1 [13]. Recent studies have also established a role for the TTG1-GL3 interaction in establishing the regular patterns of trichomes on a leaf; by sequestering TTG1 in the nucleus of trichome cells, GL3 creates a field of cells with lower TTG1 levels not competent to entering the trichome pathway [19].

**Figure 1:**
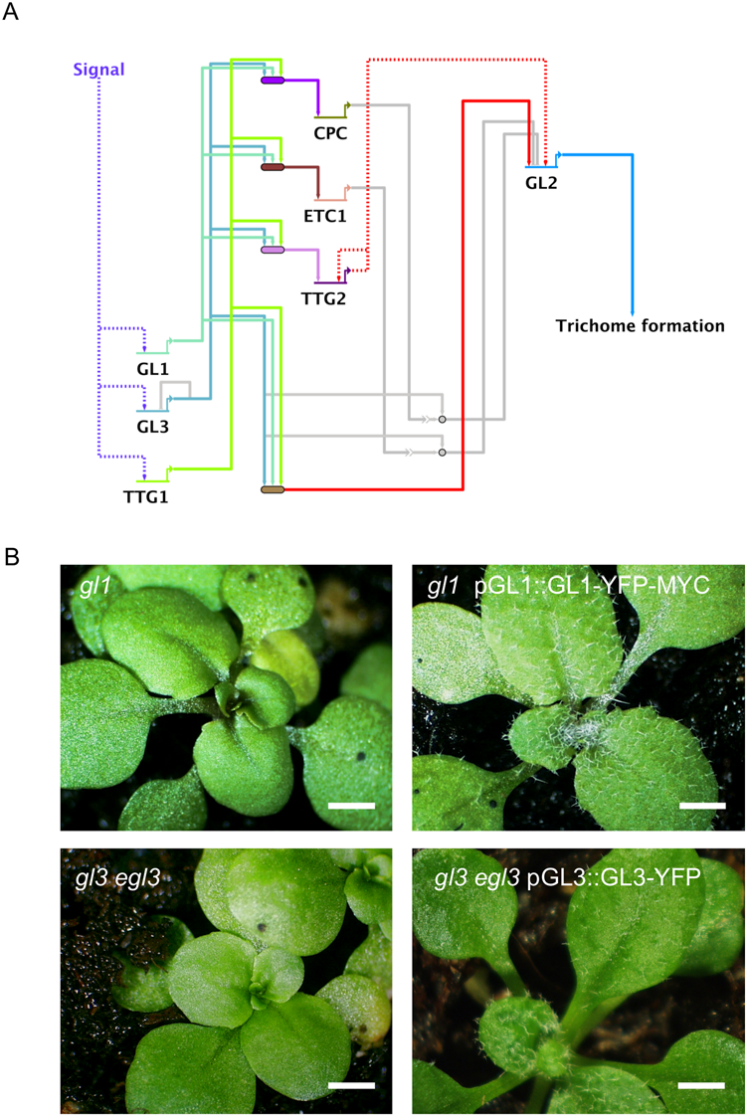
Gene regulatory network controlling trichome initiation. (A) BioTapestry [64] wire-graph representation of the regulatory circuitry involved in trichome initiation. GL3 and GL1 bind and activate the transcription of *CPC*, *ETC1*, *TTG2* and *GL2* [13]. CPC (and perhaps ETC1) can move to adjacent cells [12], and through interactions with GL3, compete for GL1/GL3 complex formation. Under these circumstances, GL3 is predicted to bind and negatively modulate its own expression [13]. Filled lines indicate direct regulation whereas dotted lines indicate indirect regulation or cases for which the regulatory mechanisms are unknown. Colored lines show active network connections in trichome cells and grey lines indicate network connections more likely to become active in epidermal cells adjacent to trichomes. (B) Complementation of the trichome phenotype of *gl3 egl3* and *gl1* mutants by pGL3::GL3-YFP and pGL1::GL1-YFP-MYC, respectively. Bar = 1 mm.

Based on the studies described above, *GL3* and *GL1* meet many of the characteristics of selector genes [20],[21] that govern the fate of a discrete niche of leaf epidermal cells (protodermal cells). So far, however, only five immediate direct targets of GL3 have been identified (Figure 1A). Three of these, TRY, CPC and ETC1, are predicted to participate in lateral inhibition, resulting in the normal trichome spacing pattern. In contrast, GL2 and TTG2 are the only two GL1/GL3 direct targets known to play a positive role in trichome formation (Figure 1A). Mutants in *gl2* and *ttg2*, however, form either small trichome primordia that fail to progress [14] or unbranched trichomes [22], respectively, suggesting that GL3 and GL1 must directly control other proteins that function in the initial stages of epidermal cell differentiation.

Here, we describe the identification of a set of genes directly regulated by GL3 and GL1. These GL3/GL1 direct targets, identified by a combination of chromatin immunoprecipitation (ChIP) methods coupled with the hybridization of whole-genome *Arabidopsis* tiling arrays (ChIP-chip) and ChIP analyses on factors known to be involved in trichome formation, include genes involved in the regulation of gene expression, in the control of endoreduplication, in metabolic functions as well as several genes with previously unknown functions. Gene expression in plants expressing the dexamethasone-(DEX) inducible GL1-GR or GL3-GR fusions indicated that many of these genes peak very rapidly (few minutes to a few hours) after GL3/GL1 induction, suggesting that they play important roles in early events associated with the differentiation of protodermal cells into trichomes. We compared changes in gene expression between wild type and *gl3* or *gl1* mutant plants, and using a meta-analysis statistical approach, we combined this data with temporal alterations in gene expression in plants expressing the GL1-GR or GL3-GR fusions. These analyses resulted in the identification of a minimal set of 513 genes associated with trichome formation. This information was combined with the GL3/GL1 direct target identification to start establishing the architecture of the trichome regulatory network.

## Results

### Genome-Wide Identification of GL3 and GL1 Binding Sites by ChIP-chip

To identify the genomic regions bound *in vivo* by the GL1 and GL3 transcription factors, we took advantage of the ability of the pGL3::GL3-YFP and pGL1::GL1-YFP-MYC transgenes to complement the trichome defect of the *gl3 egl3* and *gl1* trichome mutants (Figure 1B). We adapted chromatin immunoprecipitation (ChIP) methods coupled with the hybridization of whole-genome *Arabidopsis* tiling arrays (ChIP-chip) using antibodies against GFP (αGFP) to immunoprecipitate the chromatin fragments associated with the GL3-YFP and GL1-YFP-MYC regulators obtained from formaldehyde cross-linked green tissues of three-week old *Arabidopsis* plants. As negative controls, we utilized similar tissues from wild type *Arabidopsis* plants (i.e., not expressing pGL3::GL3-YFP or pGL1::GL1-YFP-MYC), and we performed ChIP-chip experiments with IgG on chromatin obtained from *gl3 egl3* pGL3::GL3-YFP-MYC plants. For each antibody, two independent biological replicas were performed. To identify genomic regions with a significant signal enrichment for both GL3-YFP and GL1-YFP-MYC, we utilized MAT [23], which provides a robust tool for the analysis of ChIP-chip experiments on Affymetrix tiling arrays [24]. Applying a cut-off *P*-value of 0.001, a total of 5,328 and 5,085 probes (identified by a sliding window approach using MAT, hence the value of the probes do not correspond to the raw signal values from single probes in the array, but rather to a combination of ten probes integrated through the sliding window) showed significant scores for GL1 and GL3, respectively (Table 1 and Figure S1). To identify the specific regions enriched in GL3 and GL1, we used the Integrated Genome Browser (IGB), by defining a peak as one or several probes with a significant score separated by less than 100 bp. Applying this criterion, the 5,328 probes identified as preferentially enriched in the GL1-YFP-MYC ChIP could be clustered into 680 peaks, and the 5,085 probes from GL3 into 873 peaks (Table 1). The regions recognized by GL3 and GL1 were significantly enriched (*P* = 5.5×10^−6^ for GL3 and *P* = 2.2×10^−16^ for GL1; χ^2^ test) in intergenic regions, compared with the overall distribution of the probes in the array. In contrast, both the GL3 and GL1 bound regions were significantly under-represented in coding sequences (*P* = 1.6×10^−6^ for GL3 and *P* = 2.2×10^−16^ for GL1) (Figure S2A). Enriched signals from both GL1 and GL3 were clearly located in the proximal region with respect to the transcription start site (TSS). Strikingly, the maximum enriched locations for GL1 were significantly closer to the TSS than the ones for GL3 (Figure S2B).

**Table 1:** Number of Regions Bound by GL3 and GL1.

	Number of probes/regions showing statistically significant MAT scores (*P*<0.001)		Total number of probes
	GL1	GL3	
Probes	5,328	5,085	3,039,996
Peaks	680	873	N.A.
Number of target genes	537	708	N.A.

N.A: Not available.

To identify the genes most likely corresponding to these peaks, we scanned the genome for ∼3 kb downstream of where the significant signals were located. The negative controls (ChIP carried out on wild type plants with αGFP or on pGL3::GL3-YFP/PGL1::GL1-YFP-MYC with IgG) were analyzed in a similar way, and used for subtracting the signals from the GL3 and GL1 experiments. A total of 537 and 708 genes were identified as located proximal and downstream to the GL1 and GL3 binding regions, respectively (Table 1). To validate the results from the ChIP-chip experiments in an unbiased fashion, we randomly selected 20 and 15 genomic regions identified by MAT as enriched for GL3 and GL1, respectively. A total of 14 out of 20 regions provided robust and reproducible signals in ChIP experiments using pGL3::GL3-YFP plants (Figure S3B), and 12 out of 15 for GL1 (Figure S3A), suggesting experimentally validated (rather than predicted) false positive discovery rates of 0.3 and 0.2 for GL3 and GL1, respectively.

Among the genes previously shown to be direct targets of GL3 or GL1 [12],[13], the ChIP-chip experiments identified *TTG2*, *CPC* and *ETC1* as targets for GL3, and *TRY* for GL1 (Table 2). In addition, ChIP-chip identified At5g04470 (*SIM*), At3g12280 (*RBR1*), At2g26250 (*FDH*) and At4g01060 (*CPL3*) as GL3 direct targets and At1g63910 (*AtMYB103*) as a target of GL1 (Table S1 and Figure S5). *SIM*, *RBR1*, *FDH*, *CPL3* and *AtMYB103* are among approximately 70 genes that have been identified as participating in various aspects of trichome initiation, branching, morphology and distribution (Table S1). Taken together, these results suggest that the ChIP-chip experiments have been successful in identifying putative targets for the trichome regulators, GL3 and GL1.

**Table 2:** Summary of Genes identified as GL3 or GL1 Direct Targets.

AGI	Gene name	ChIP-chip GL1	ChIP-chip GL3	ChIP in GL3-YFP	ChIP in GL1-YFP
*Previously identified GL3/GL1 Direct Target*					
*At1g79840*	GL2	−	−	+	+
*At1g01380*	ETC1	−	+	+	+
*At2g46410*	CPC	−	+	+	+
*At2g37260*	TTG2	−	+	+	+
*At5g53200*	TRY	+	−	+	+
*At5g41315*	GL3	−	−	+	−
*GL3/GL1 Direct Target Identified by ChIP*					
*At5g52510*	SCL8	+	+	+	+
*At3g50800*	TGS-like domain protein*	+	+	+	+
*At5g28350*	WD repeat protein*	+	+	+	+
*At3g50790*	LEA protein	+	+	+	+
*At4g20960*	Cytidine/deoxycytidylate deaminase family protein	+	+	+	+
*At1g77670*	Aminotransferase	+	+	+	+
*At3g10113*	MYB transcription factor	+	+	−	−
*GL3/GL1 Direct Target Identified by ChIP-chip*					
*At5g04470*	SIM	−	+	+	+
*At3g12280*	RBR1	−	+	+	+
*At2g26250*	FDH	−	+	+	+
*At4g00480*	MYC1	−	−	+	+
*At4g01060*	CPL3	−	+	+	+
*At1g71030*	MYBL2	−	−	+	+
*At1g63910*	MYB103	+	−	−	−

*: Predicted by PHYRE.

### GL3 and GL1 Target Together a Small Set of Genes

**Figure 2:**
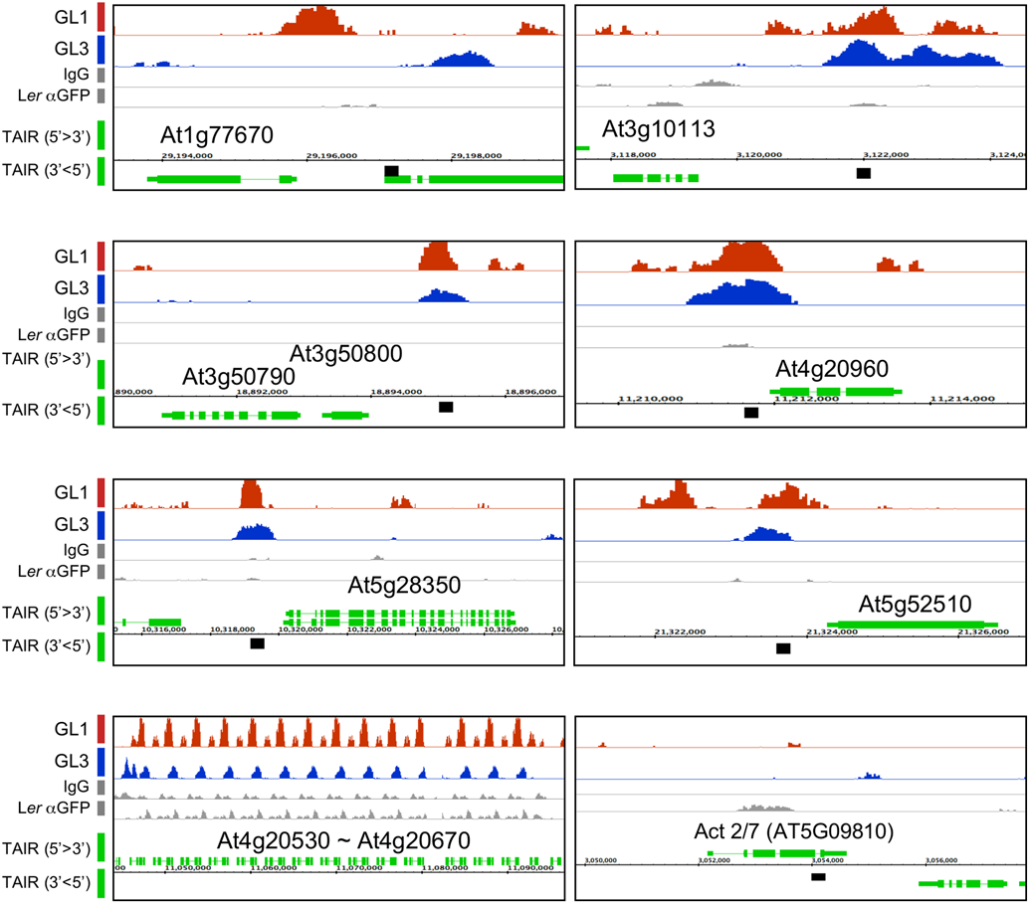
Genome-wide identification of GL1 and GL3 associated regions. Signal enrichment location displayed using IGB of GL1 (orange) and GL3 (blue) associated regions as well as signals obtained from the negative ChIP-chip controls using IgG and αGFP on L*er* wild type plants (gray). The gene annotation, shown in green, was obtained from TAIR (http://www.arabidopsis.org/index.jsp). Large boxes correspond to exons; small boxes to untranslated regions and lines to introns. Gene orientations are indicated on the left side of the picture. The black boxes indicate the region utilized for ChIP-PCR verification.

**Figure 3:**
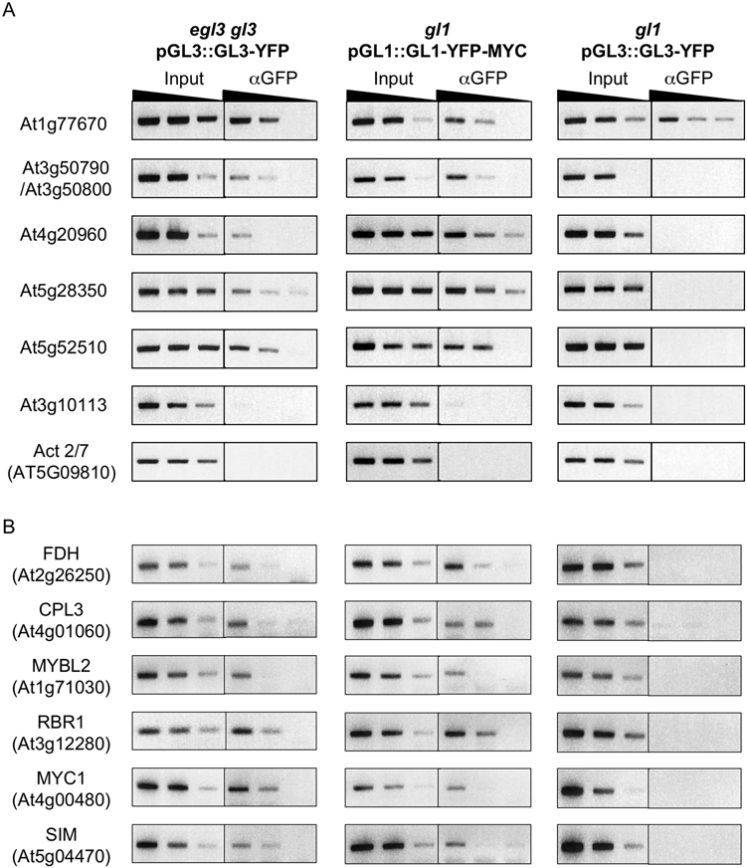
Validation of GL1 and GL3 direct target genes. (A) Semi-quantitative PCR of ChIP experiments performed on *gl3 egl3* pGL3::GL3-YFP, *gl1* pGL1::GL1-YFP-MYC or *gl1* pGL3::GL3-YFP plants of genes selected by ChIP-chip. Serial 4-fold dilutions used for PCR are represented by the black triangles. PCR were performed on regions 500 bp upstream from the TSS, except for Act 2/7, for which PCR was performed on the 5′UTR. (B) Semi-quantitative PCR of ChIP experiments performed on *gl3 egl3* pGL3::GL3-YFP, *gl1* pGL1::GL1-YFP-MYC or *gl1* pGL3::GL3-YFP plants of trichome genes (Table S1) identified as GL3 and GL1 targets (Figure S5). Serial 4-fold dilutions used by PCR are represented by the black triangles. PCR were performed on regions 500 bp upstream from the TSS, except for Act 2/7, for which PCR was performed on the 5′UTR.

We predicted that at least some of the genes involved in trichome initiation should be targets of both GL3 and GL1, similar as we demonstrated previously for *GL2* and *TTG2* [12],[13]. A total of 20 genomic regions (corresponding to 21 genes) showed a significant enrichment for both GL3 and GL1 (Figure 2). One of these regions corresponded to a tandem repeat of 14 genes corresponding to *At4g20530* - *At4g20670* on which a lower signal was also detected in the ChIP-chip negative control (Figure 2, see *At4g20530* - *At4g20670*). Thus, for the subsequent studies, we will not focus on genes within this tandem arrangement since we are not confident on whether the signal observed with GL3 and GL1 is real or not. It is not uncommon, however, for simple tandem repeats to be associated with false positives in ChIP-chip experiments [24]. We individually analyzed by regular ChIP the seven remaining genes identified as putatively bound by both GL3 and GL1. Six of the seven genes (*At1g77670*, *At3g50790*/*At3g50800*, *At4g20960*, *At5g28350* and *At5g52510*) were confirmed as recognized by both GL3 and GL1, and one (*At3g10113*) showed no binding by either regulator in the promoter region tested (Table 2 and Figure 3A).

*At5g52510* corresponds to *SCL8*, a divergent member of the GRAS family of regulatory proteins [25]. *At3g50800* and *At5g28350* are annotated as unknown ‘expressed proteins’ in TAIR. However, the protein structure threading program, PHYRE (http://www.sbg.bio.ic.ac.uk/phyre/html/index.html) predicted them as TGS-like domain and WD-repeat proteins, respectively (Figure S4A). While WD-repeats are often associated with protein-protein interaction [26], the function of the TGS domains, named after ThrRS, GTPase, and SpoT, is less well know, but was proposed to bind nucleotides [27]. PHYRE also predicted At5g28350 to contain a motif conserved in the yeast RIC1 protein (the RIC1 domain, Figure S4A), perhaps involved in the transport of endosome-derived vesicles to the Golgi network [28]. *At3g50790* encodes a putative hydrolase, which belongs to the late embryogenesis abundant (LEA) proteins [29], and which is broadly expressed in green tissues at most developmental stages (Figure S4B, C). *At4g20960* is annotated in TAIR as diaminohydroxyphosphoribosyl aminopyrimidine deaminase (EC 3.5.4.26), which catalyzes the second step in riboflavin biosynthesis. *At1g77670* is predicted to encode pyridoxal phosphate dependent transferase involved in the biosynthesis of amino acids and amino acid-derived metabolites [30].

Previously, we described three mechanisms by which GL3 could bind and presumably control, target gene expression. The first mechanism requires the presence of a functional GL1 protein to tether GL3 to the *GL2*, *CPC* and *ETC1* gene promoters [13]. Working by the second mechanism, GL3 can bind the *TRY* promoter independently of GL1, although both GL3 and GL1 are necessary for *TRY* activation. By the third mechanism, GL3 binds and regulates transcription independently of GL1, as we showed for the negative auto-regulation of *GL3* [13]. Thus, we investigated which of these mechanisms might be at play in the control of the six newly identified GL3 and GL1 direct targets. Towards this goal, we expressed the pGL3::GL3-YFP transgene in the *gl1* mutant, as previously described [13], and performed ChIP experiments (with αGFP). For At3g50790/At3g50800, At4g20960, At5g28350 and At5g52510, the binding of GL3 required the presence of GL1, suggesting that the regulation of these genes occurs by the first mechanism, as is the case for *GL2*, *TTG2*, *CPC* and *ETC1*. Only in the case of At1g77670, the binding by GL3 was independent of GL1 (Figure 3A, compare *gl3 egl3* pGL3::GL3-YFP and *gl1* pGL3::GL3-YFP), despite the fact that GL1 also bound this promoter (Figure 3A, *gl1* pGL1::GL1-YFP-MYC). Together, these results identify a set of new direct targets for both GL3 and GL1, corresponding to genes of known and unknown functions likely involved in early stages of trichome initiation.

### A Subset of Genes with Trichome Functions Correspond to Direct Targets of GL3 or GL1

Despite being an outstanding tool for the identification of direct targets for transcription factor, ChIP-chip has a notorious false negative rate (*i.e.*, real positives that fail to be identified) [24]. Thus, we took a complementary approach to identify additional putative direct targets of GL3 and GL1. Based on the effect of mutations, ∼70 genes have been identified as participating in various aspects of trichome initiation, pattern formation, endoreduplication and morphology (Table S1). Only six of these genes (*GL2*, *TTG2*, *GL3*, *TRY*, *ETC1* and *CPC*) had been previously identified as GL3-GL1 direct targets [12],[13], and our ChIP-chip experiments identified five more (*SIM*, *RBR1*, *FDH*, *CPL3* and *AtMYB103*) as targets of GL3, GL1 or both (Figure 3B and Table 2).

Thus, we asked whether genes described as involved in trichome morphogenesis might be direct targets for GL3 and/or GL1, by testing the presence of a region spanning a 500 bp upstream of the TSS for each of these candidate genes in ChIP experiments performed on *gl3 egl3* pGL3::GL3-YFP or *gl1* pGL1::GL1-YFP-MYC transgenic plants. Representative examples of the results of these experiments are shown in Figure S5 and the data is summarized as part of Table 2 and Table S1. Interestingly and highlighting the false negative discovery rate of ChIP-chip experiments, *SIM*, *RBR1*, *CPL3* and *FDH*, which were only found in the ChIP-chip experiments with GL3-YFP, showed reproducible tethering of both GL3 and GL1 to the corresponding promoters in ChIP assays, suggesting that they should be added to the list of shared direct targets of GL3 and GL1 (Table 2). *MYC1*, which did not come up in the ChIP-chip experiments as either a target of GL3 nor of GL1, showed robust binding by both regulators in conventional ChIP assays (Figure 3B). In contrast, *MYB103*, a gene involved in endoreduplication [31] and identified as a GL1 target by ChIP-chip, could so far not be validated by ChIP as a GL1 target, thus *MYB103* will not be further considered in this study. Taken together, these results expand to 19 the set of genes directly regulated by both GL3 and GL1. GL3 and GL1 participate in complexes with other R2R3-MYB and bHLH proteins, respectively. For example, GL1 interacts with EGL3 and MYC1, and GL3 interacts with MYB23 [32]. Thus, genes regulated just by GL3 or GL1 could be very interesting in understanding how different MYB/bHLH complexes target distinct sets of target genes.

### Identification of Genes Regulated by GL3 and GL1

**Figure 4:**
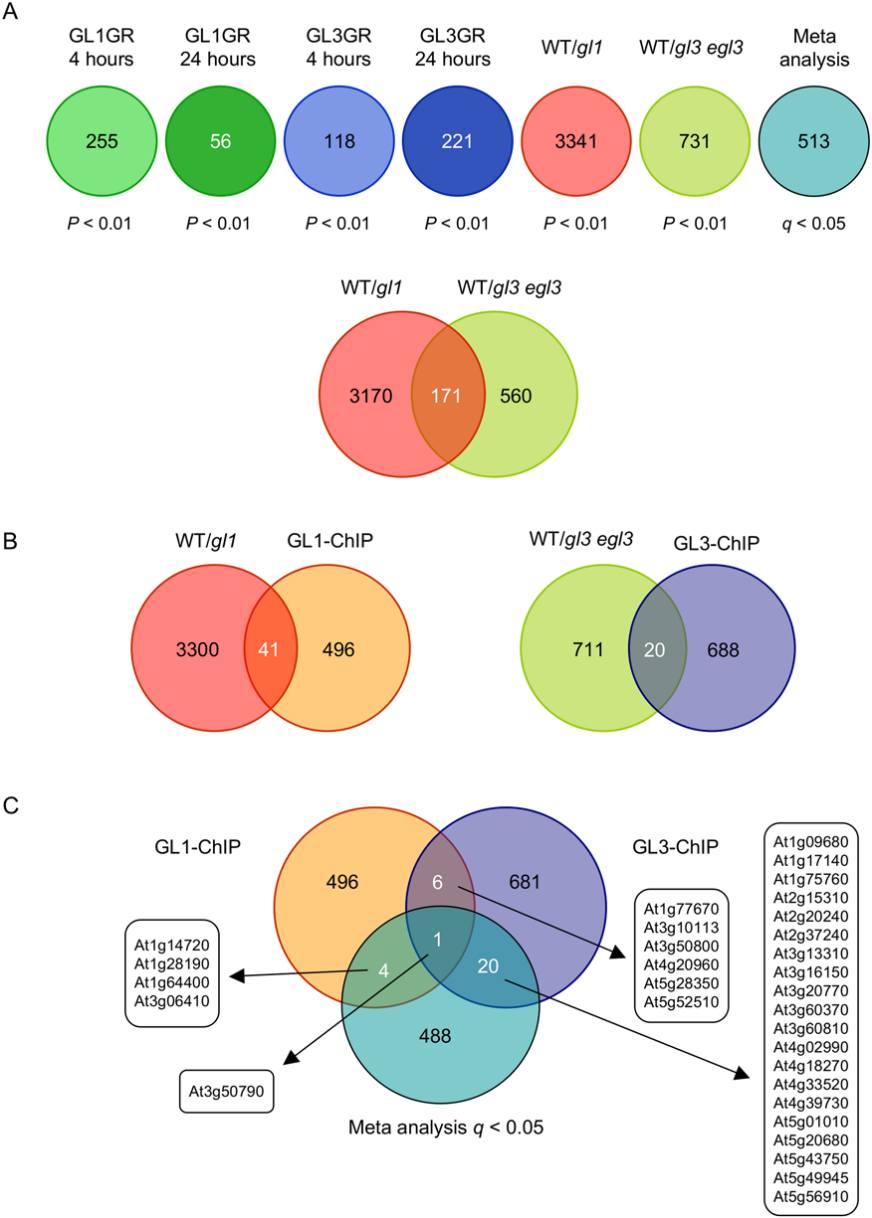
Genome-wide expression changes induces by GL3 or GL1. (A) Venn diagram representation of the genome-wide alterations in mRNA accumulation in *gl1* pGL1::GL1-GR plants induced 4 (GL1GR 4 hours) or 24 hrs with DEX (GL1GR 24 hours), *gl3 egl3* pGL3::GL3-GR plants induced 4 (GL1GR 4 hours) or 24 hrs with DEX (GL1GR 24 hours), differentially expressed genes between wild type and the *gl1* mutant (WT/*gl1*) or between wild type and the *gl3 egl3* mutant (WT/*gl3 egl3*). Numbers inside the diagrams represent the total umber of genes affected at the indicated *P* value. Genes estimated to be affected by GL1 and GL3, forming a minimal “Trichome genes” set, were deduced using a meta-analysis statistical approach (see Materials and Methods). (B) Venn diagrams indicate the number of genes that were affected by two or more of the contrasts described in (A). (C) Overlap between gene expression and ChIP-chip analyses. The AGI numbers for the genes in each intersection are indicated.

To investigate the role of GL3 and GL1 on the expression of trichome genes, we took two complementary strategies. In the first approach, we performed genome-wide gene expression analyses using Affymetrix ATH1 arrays with RNA extracted from green tissues obtained from 14 days-old wild-type, *gl1* or *gl3 egl3* seedling. Statistical analysis performed on biological triplicates revealed that 3,341 genes were differentially expressed in *gl1* plants, compared to wild type, and 731 genes were differentially expressed in *gl3 egl3* plants, compared to wild type (Figure 4A). Out of the 3,341 genes, 41 genes were identified in the ChIP-chip experiments as GL1 direct targets, and out of the 731 genes, 20 genes were found to be direct targets of GL3 (Figure 4B).

Since trichome formation progresses in parallel with leaf development, the plants used for these expression analyses contain leaf hairs at all possible stages, making it difficult to determine at what stage of trichome formation the GL3/GL1 targets may function. As a first approximation to identify GL3/GL1 targets participating in early stages of trichome initiation (likely under-represented in the previous analyses), the second approach took advantage of plants expressing translational fusions of GL3 or GL1 with GR, driven by the corresponding promoters (pGL3::GL3-GR and pGL1::GL1-GR). As previously described, *gl3 egl3* pGL3::GL3-GR and *gl1* pGL1::GL1-GR plants accumulate trichomes only in the presence of dexamethasone (DEX) [13]. Genome-wide expression analyses were performed on *gl3 egl3* pGL3::GL3-GR and *gl1* pGL1::GL1-GR plants at 4 hrs and 24 hrs after DEX induction, and compared with Mock-treated plants. Statistical analyses resulted in the identification of 255 and 56 genes affected by GL1-GR induction at 4 and 24 hrs, respectively. Similar analyses performed on pGL3::GL3-GR plants resulted in the identification of 118 and 221 genes affected at 4 and 24 hrs, respectively (Figure 4A). Interestingly, the identity of the genes affected by GL3 and GL1 after 4 or 24 hrs of induction were strikingly different (Figure S6), suggesting a clear distinction in the gene functions necessary for earlier and later stages of trichome formation. The lower number of genes affected by GL1-GR at 24 hrs, compared with GL3-GR at 24 hrs, is in agreement with models suggesting that the function of GL1 is primarily limited to earlier stages of trichome development, while the effects of GL3 extend into later stages, including branch formation [33].

To establish a minimal set of genes uniquely associated with the formation of trichomes controlled by GL1 and/or GL3, statistical meta-analyses were performed. Briefly, using the *P* value statistics obtained from the six microarray experiments (*gl1* versus wild type, *gl3 egl3* versus wild type, *gl1* pGL1::GL1-GR 4 and 24 hrs DEX induction, and *gl3 egl3* pGL3::GL3-GR 4 and 24 hrs DEX induction; all experiments done in biological duplicates or triplicates, see Materials & Methods), *q*-values were calculated as described [34] (see Materials & Methods). This analysis resulted in the identification of a minimal set of 513 genes (*q*<0.05) associated with the GL1/GL3 induction of trichomes. Based on Gene Ontology (GO) analyses, this group of genes showed a significant enrichment in (1) metabolism, (2) energy, (3) protein fate, (4) cellular communication and signal transduction mechanism, (5) cell rescue, defense and virulence, (6) interaction with the environment, (7) systemic interaction with the environment, (8) development and (9) subcellular localization (Figure S7). These findings define a minimal set of 513 genes associated with trichomes, a set that is hierarchically positioned downstream of GL3, GL1 or both. Only 4 and 20 genes were found to overlap between the meta-analysis and GL1 or GL3 ChIP-chip experiments, respectively (Figure 4C). This may reflect GL1 and GL3 bind many promoters without a significant effect on their expression, as has been found to be the case for some transcription factors in animals [35], and that many of the meta-analysis identified genes correspond to indirect targets of GL3/GL1.

### Temporal Expression of the GL3/GL1 Direct Targets during Trichome Initiation

**Figure 5:**
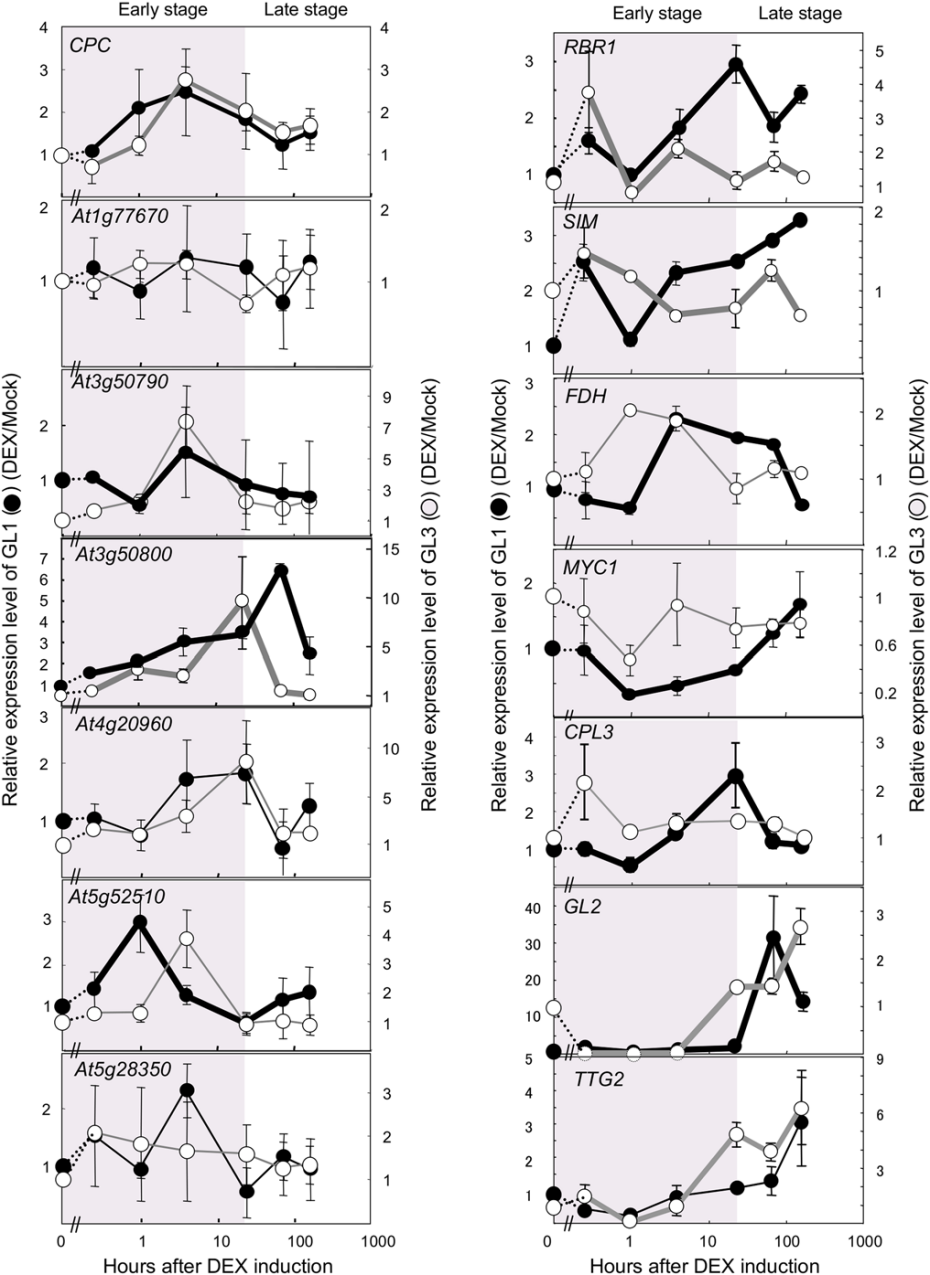
Expression of a select set of GL3 and GL1 direct targets after DEX induction of GL3-GR or GL1-GR. qRT-PCR experiments performed on mRNA obtained from *gl1* pGL1::GL1-GR or *gl3 egl3* pGL3::GL3-GR seedlings DEX- or Mock-treated for various times (15 min, 1 hr, 4 hrs, 24 hrs, 3 days and 7 days). It should be noticed that the y-axis is drawn in linear-scale whereas the x-axis is in log-scale. Black circles correspond to expression in *gl1* pGL1::GL1-GR plants and white circles to expression in *gl3 egl3* pGL3::GL3-GR plants. For each time-point, the ratio between the DEX and Mock treatment was calculated. For clarity purposes, the pre-induction condition (time = 0) is shown, and corresponds to no induction (ratio = 1). Induction times, corresponding to early stages of trichome development (0–24 hrs), are indicated shaded gray. For each time point, biological triplicates were collected and the error bar indicates the standard error. Thick lines correspond to gene expression profiles for which ANOVA statistical analyses suggested a significant difference (*P*<0.05). Thin lines indicate cases for which statistical significance could not be supported to biological variation between the triplicates.

To further delineate the specific stages during trichome formation at which the immediate direct targets of the GL3/GL1 complex function, we explored their expression by quantitative real-time RT-PCR (qRT-PCR) in *gl3 egl3* pGL3::GL3-GR and *gl1* pGL1::GL1-GR plants at various times after DEX induction (Figure 5). While in some cases, biological variation between the triplicates used in these experiments interfered with statistical significance tests, specific trends in the response of the target genes to GL3 and GL1 induction become evident when looking at overall patterns. Consistent with the *gl2* and *ttg2* mutant phenotypes, suggesting functions after trichome initiation, perhaps during the growth and maturation of a trichome primorida, *GL2* and *TTG2* expression peak 24–48 hrs following GL3 or GL1 induction. In contrast, the peak in *CPC* mRNA accumulation occurs 1–4 hrs after GL3 or GL1 induction. Similar to *CPC* and suggesting functions needed very early in trichome initiation, *At3g50790*, *At5g52510*, *At5g28350*, *MYC1* and *FDH* show mRNA accumulation peaks within 10 hrs of GL3 or GL1 induction. The increase in *At5g52510* mRNA accumulation controlled by GL3 is slightly delayed, compared with GL1, and the steady-state mRNA levels of *At5g28350* are primarily affected by GL1, with a lesser effect by GL3 despite it being recruited to the *At5g28350* promoter (Figure 3A). *RBR1* and *SIM* show very similar mRNA accumulation patterns, with an early induction peak within 15 minutes, and a later peak after 24 hrs of GL3 and GL1 induction. This later peak is also observed for *At3g50800* and *At4g20960*.

ChIP-chip and ChIP analyses did not permit us to determine whether the GL3/GL1 recruitment to the intergenic region of *At3g50790/At3g50800* (Figures 2 and 3) regulated the expression of one gene, the other or neither. It is evident from the qRT-PCR experiments that GL3 and GL1 modulate the mRNA accumulation of both *At3g50790* and *At3g50800* in different ways. While *At3g50790* mRNA peaks at around 4 hours after GL3 and GL1 induction, the expression of *At3g50800* peaks at around 24 hrs (Figure 5). The TSSs for these genes are separated by just 320 bp, and the genes are oriented in a head-to-head organization (Figure S8). Taken together, these results show that GL3 and GL1 direct targets peak early during trichome formation, with a clear distinction between very early genes (<10 hrs) or later genes (>24 hrs), suggesting that the corresponding gene products are similarly required within those particular developmental windows.

## Discussion

In this study, we have taken a comprehensive systems approach combining ChIP-chip, candidate gene approaches and genome-wide expression analyses to identify genes regulated by the trichome regulators GL3 and GL1, and to investigate the architecture of the gene regulatory network responsible for the differentiation of epidermal cells into trichomes in *Arabidopsis*. Our results suggest novel regulatory functions for GL3 and GL1 highlighted by the identification of a set of previously unidentified GL3/GL1 immediate direct targets. Some of these targets express before any of the morphological changes associated with epidermal cell differentiation, suggesting very early functions in the trichome developmental program. Others peak after the first changes are evident, suggesting a need for the progression from trichome initials into mature trichomes. The integration of this information provides a first blueprint for the regulatory network involved in trichome formation.

### Identification and Expression of New GLI and GL3 Immediate Direct Target Genes

Previous studies had identified just six direct targets for the GL3/GL1 trichome regulators, from which GL2 and TTG2 were the only known positive regulators (Table 2). Yet, the phenotype of *gl2* and *ttg2* mutations (trichomes arrested as small protuberances) indicated that, while the *GL2* and *TTG2* gene products are important for the maturation of trichome initials, they probably did not function during early trichome initiation steps. Thus, we combined two approaches towards the identification of novel GL3/GL1 direct targets: ChIP-Chip experiments using GFP-tagged proteins and candidate gene approaches, taking advantage of the rich collection of trichome mutants available (Table S1). We attempted to identify GL3 and GL1 direct targets using plants harboring the corresponding GR fusions [12],[13], by comparing genes affected by DEX in the presence and absence of the protein synthesis inhibitor cycloheximide (CHX), but CHX often masked the effects of DEX, making the approach, at least for this particular set of regulators, impractical. Table 2 lists all the so far known GL3/GL1 direct targets and the evidence supporting it. Among the new GL3/GL1 direct targets, our studies identified *SIM* (*SIAMESE*), *RBR1* (*RETINOBLASTOMA RELATED1*), *FDH* (*FIDDLEHEAD*), *MYC1*, *MYBL2* and *CPL3* (*CAPRICE-LKE MYB3*) (Table 2).

*FDH* encodes a β-ketoacyl-CoA synthase related protein, which has been implicated in modifying the properties of the cuticule, preventing epidermal fusions [36]–[38]. Consistent with a role in trichome formation, *fdh* mutants show a significant reduction in the number of trichomes [37]. Suggesting a participation of FDH and cuticule functions early in trichome formation, *FDH* mRNA levels peak at around 4 hrs after GL3/GL1 induction (Figure 5).

**Figure 6:**
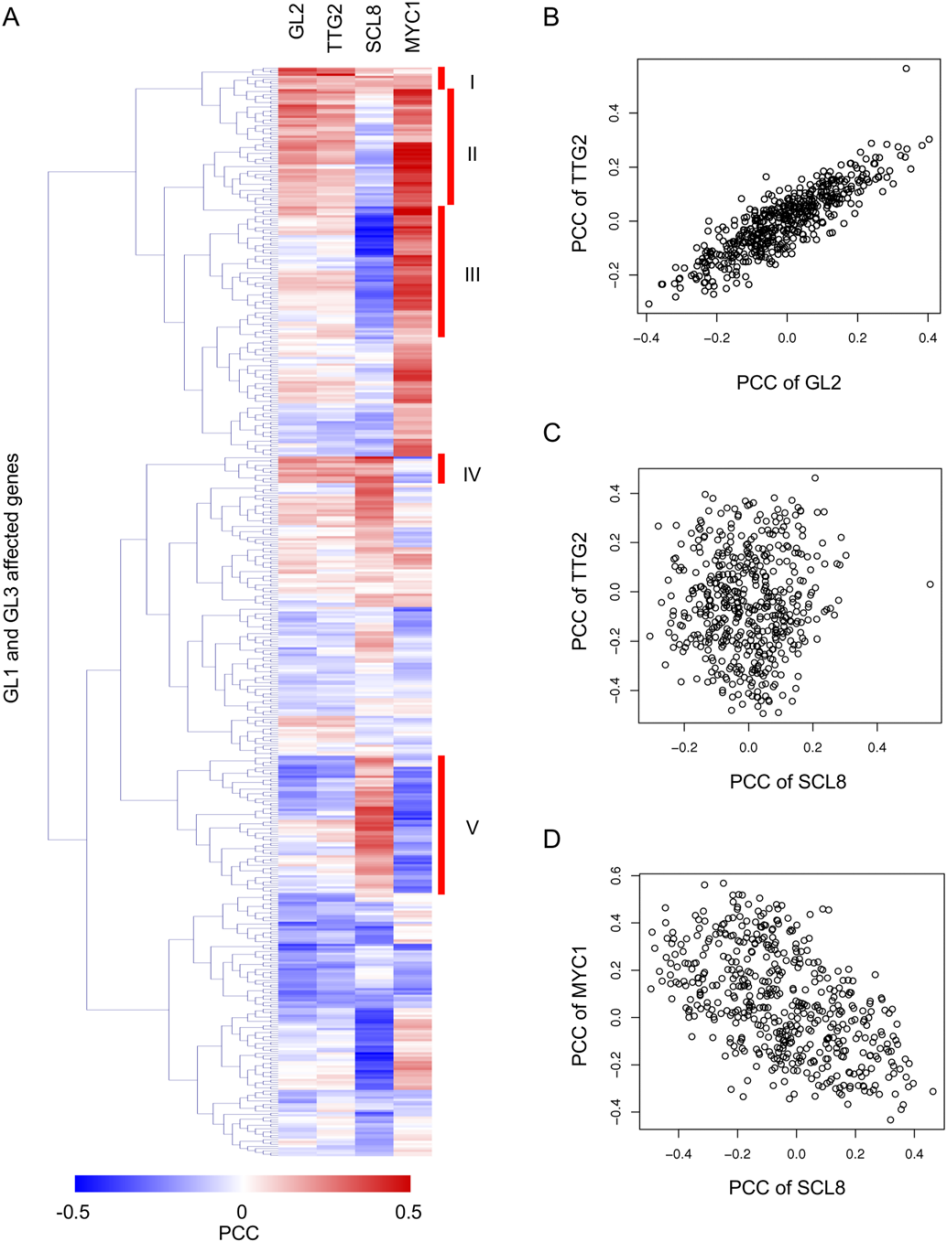
Co-expression of the minimal set of 513 trichome genes with GL2, TTG2, SCL8 and MYC1. (A) The heat map shows a distribution of PCC values after hierarchical clustering. Red and blue colors indicate positive and negative PCC values, respectively. Clusters I–V, discussed in the text, are indicated with red bars on the right side of the heat map. (B–D) Scatter plots comparing the PCC valued of all genes in the trichome set with (B) *TTG2* and *GL2*, (C) *TTG2* and *SCL8*, and (D) *MYC1* and *SCL8*.

*MYC1* encodes a bHLH factor [39] closely related to GL3 and EGL3 [40]. QTL analyses implicated MYC1 in controlling trichome numbers [41],[42]. Both GL3 and GL1 bind the *MYC1* promoter, and the tethering of GL3 requires the presence of GL1 (Figure 3B), suggesting similar mechanisms for *MYC1* regulation by GL3/GL1 as for *GL2*, *CPC* and *TTG2*. *MYC1* mRNA accumulation follows a pattern different from other regulators: It drops 1 hr after GL3 and GL1 induction to then increase back, earlier for GL3 than for GL1 (Figure 5). Thus, our results suggest the existence of a regulatory motif in which *MYC1* mRNA accumulation is partially controlled by GL3/GL1 (Figure 6). Since MYC1 was shown to interact with GL1 and other related R2R3-MYB factors [32], it is possible that the regulation of MYC1 by GL1/GL3 represents a feedforward network motifs, perhaps participating in amplifying signals for trichome initiation, or maybe involved in switching the targets from a GL1/GL3 complex to a GL1/MYC1 (or MYB23/MYC1) complex.

*CPL3* (*CAPRICE-LKE MYB3*) and *MYBL2*, similar to *CPC*, *ETC1* and *TRY*, encode single repeat MYB proteins [43]–[45]. As *CPC* but distinct from *TRY* [13], the *in vivo* recruitment of GL3 to promoter sequences in *CPL3* and *MYBL2* requires GL1, which is also tethered to DNA (Figure 3B). The expression of *CPL3*, however, is controlled with different kinetics by GL3 and GL1. In the *gl3 egl3* pGL3::GL3-GR plants, *CPL3* peaks within 15 min of DEX treatment, whereas GL1 induces its expression around 12 hrs (Figure 5).

MYBL2 has been primarily implicated as a negative regulator of anthocyanin biosynthesis [43],[44], yet *MYBL2* over-expression suppressed *Arabidopsis* trichome formation [46]. Our results, exposing MYBL2 as a GL3/GL1 direct target, further highlight its function in trichome formation. The results presented here show that GL3 and GL1 directly activate the expression of most of the known single MYB repeat inhibitors of trichome formation, including *CPC*, *ETC1*, *CPL3*, *MYBL2* and *TRY*. These single repeat MYB proteins are conserved in sequence and have been predicted to have similar functions, thus it remains to be determined why several of them need to be directly controlled by GL1 and GL3.

*SCL8* encodes a GRAS family transcription factor and *SCL8* mRNA levels peak sharply within the first few hours of GL3 or GL1 induction, to then level off at quantities similar as found in the absence of the regulators (Figure 5), suggesting a need for *SCL8* function at early stages during trichome initiation. The function of *SCL8* remains unknown. However, similar to the formation of trichomes, the initiation of axillary meristems is controlled by the action of bHLH and MYB transcription factors, leading to the speculation that similar regulatory motifs might participate in the control of these two processes [47]. Axillary meristem formation involves the *LAS (LATERAL SUPRESSOR)* GRAS family member [48]. Thus, the identification of *SCL8* as a GRAS family member involved in trichome formation further expands similarities between the regulation of these two processes. A recent study reported the analysis of genes differentially expressed in trichome by exploring the transcriptome of dissected trichomes [49]. In agreement with our results, *SCL8* is among the genes described in this study as displaying increased expression in trichomes.

In addition, our studies identified several GL3/GL1 direct target genes with unknown functions (Table 2). Through the utilization of structure-prediction programs, some specific domains were identified in the encoded proteins, which will facilitate their functional characterization and participation in trichome formation. We also found many genes that are direct targets of either GL1 or GL3, but not of both together (Figure S5 and Table 2). It is possible that the regulation of those genes involves other MYB-bHLH complexes, such as GL1-EGL3 or MYB23-GL3. Indeed, based on our results and the observation that MYB23 participates in later stages of trichome morphogenesis [11], we speculate that GL3 direct targets such as *BRK1* and *DIS1*, which function in trichome morphogenesis, and *CDKA;1* and *CYCA2;3*, likely involved in maintaining endoreduplication, are targeted by the GL3-MYB23 complex (Table S1 and Figure S6). *MYB23* was not identified as a target of GL3 or GL1 in ChIP experiments, nor was *MYB23* expression significantly affected by these regulators in either the DEX induction experiments, or the genome-wide expression analyses comparing wild-type and *gl1* or *gl3 egl3* mutant plants (not shown).

### GL3/GL1 Regulate the Endocycle by Directly Controlling *SIM* and *RBR1* Expression

Cell differentiation is often associated with a change from mitotic cell division to endoreduplication [50]. This is also the case for trichomes, which show an average DNA content of 32 C [5]. During the initial stages of differentiation of protodermal cells into trichomes, the first phenotypic change that anticipates trichome appearance is an enlargement of the nucleus, corresponding to the initiation of the endocycle [5]. GL3 was previously implicated in the control of endoreduplication [5], yet the mechanisms associated with the early cellular reprogramming associated with the switch from mitosis to the endocycle remain unknown. The identification of *SIM* and *RBR1* as immediate direct targets of GL3/GL1 provides clues on the earliest molecular mechanisms associated with this switch. *SIM* encodes a small protein with a region of similarity to cell cycle inhibitor proteins and which interacts with D-type cyclins and cyclin-dependent kinases (CDKs), such as CDKA;1 [51]. In *sim* mutants, multicellular trichomes and trichome clusters form [52]. Consistent with *SIM* being a direct target of GL3, the levels of *SIM* expression in *gl3 egl3* plants were found to be very significantly reduced [51]. Our results, highlighting a role of GL1 in the regulation of endoreduplication early during trichome initiation, is consistent with the recent identification of *sim* mutant allele as a modifier of the trichome phenotype of an allele of GL3 (*gl3-sst*) impaired in its ability to interact with GL1 [9],[53]. RBR1, through its interaction with members of the E2F family of transcription factors, regulates the balance between cell division and the endocycle, and the conditional inactivation of *RBR1* results in trichomes with altered morphologies, which include more branches [54]. Thus, while SIM participates in activating the endocycle and repressing cytokinesis during trichome formation, RBR1 is likely to restrict the number of endocycles associated with normal trichome morphogenesis. Interestingly, *SIM* and *RBR1* show very similar mRNA accumulation patterns after induction of GL3/GL1 function (Figure 5). The mRNA for both genes peaks very early (15 minutes) after the treatment with DEX, followed by a decrease that suggests rapid mRNA turnover, to increase again 10–24 hrs after GL3/GL1 induction. Our findings provide the first evidence directly implicating GL3/GL1 in the control of endoreduplication very early after the trichome initiation program has been triggered. In addition, they suggest that the initial steps in the switch from mitosis to the endocycle involve a two-pronged strategy: the inhibition of CDKs by SIM triggering the endocycle, and the inhibition of E2F by RBR1, restricting the number of endocycle rounds.

### Co-Expression Profiling Reveals Hierarchical Structure for Trichome Regulatory Network

One of the questions that this study intended to answer is whether the function of GL3 and GL1 is solely channeled through TTG2 and GL2, the only two positive regulators previously known to be regulated by the trichome regulators. Our analyses indicate, however, that this is not the case, and that at least two additional transcription factor genes, *SCL8* and *MYC1* (Table 2), are direct targets of the GL3/GL1 complex. In addition, genome-wide expression analyses, comparing genes differentially expressed between wild type and *gl3 egl3* or *gl1-1* plants, as well as those affected by DEX in *gl3* pGL3::GL3-GR and *gl1* pGL1::GL1-GR plants, implicated a minimum set of 513 genes (“trichome genes”) as directly or indirectly controlled by GL3 or GL1. Thus, starting from the assumption that the trichome regulatory module has a hierarchical layout with the GL3/GL1 regulators at the top (first tier regulators), we investigated the relationship between the “trichome genes” and the corresponding regulators.

**Figure 7:**
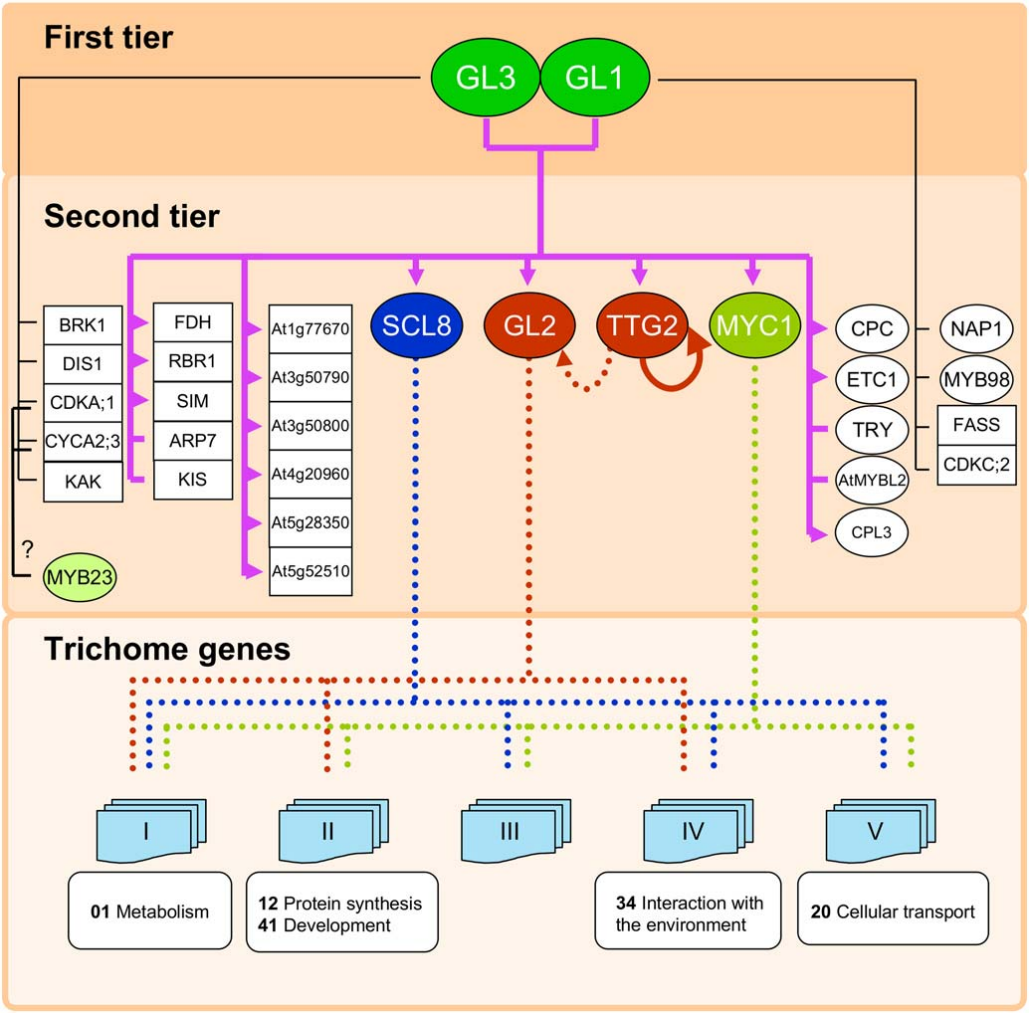
Regulatory motifs controlled by GL1 and GL3. Circle and squares indicate the nodes of the directed graph representing the regulatory network, and correspond to transcription factors, and enzymes or structure proteins, respectively. Filled lines indicate the edges of the regulatory network, and correspond to direct interactions between transcription factors and the corresponding target genes. The color of the lines originating in the GL3/GL1 first tier regulators indicate whether they correspond to direct targets of GL3 and GL1 (purple), GL3 alone (or perhaps with MYB23, black) or GL1 alone (black). Lines with arrowheads indicate activation by GL3/GL1 whereas lines without arrowheads mean gene regulation is uncertain. The dotted lines derived from the second tier regulators SCL8, GL2, TTG2 and MYC1 correspond to genes from the minimal trichome set most likely downstream of each of the regulators. Boxes shown in the “Trichome genes” tier indicate the corresponding functional classifications. Numbers in bold represent the gene functions classified by FunCat (http://mips.gsf.de/proj/funcatDB/search_main_frame.html). All the genes shown here are described in more detail as part of Table S1.

*GL2*, *TTG2*, *MYC1* and *SCL8* all correspond to regulatory factors directly controlled by GL3/GL1, hence constitute second tier regulators (Figure 7). The other identified GL3/GL1 direct targets (Table 2) are either not predicted to correspond to transcription factors (Figure 7), or modulate the activity of the GL3/GL1 complex, as is the case of the single MYB repeat proteins, and thus feed-back control tier 1 regulators. Thus, the 513 “trichome genes”, if they are not direct targets of GL3/GL1, they must be downstream of one or several of the second tier regulators.

To determine the relationship of the “trichome genes” with each one of the second tier regulators, we investigated which genes were co-expressed more tightly with each of the regulators, using Pearson's Correlation Coefficient (PCC) obtained from ATTED-II (http://www.atted.bio.titech.ac.jp/), and surveying the expression data generated by AtGenExpress [55]. Interestingly, the distribution of PCC scores of “trichome genes” with *GL2* and *TTG2* was almost identical, as evidenced by heat-maps of PCC values after hierarchical clustering (Figure 6A, B). In contrast, there was no significant overlap in the “trichome genes” co-expressed with *SCL8* and those co-expressed with *GL2* or *TTG2* (Figure 6A, C), although a weak negative correlation between the genes co-regulated with *SCL8* and those co-regulated with *MYC1* was observed (Figure 6A, D). These results suggest that SCL8 controls a very different set of trichome genes than GL2, TTG2 and MYC1.

Based on the hierarchical clustering of coexpression values with the GL2, TTG2, SCL8 and MYC1 regulators, the “trichome genes” were classified into 5 arbitrary groups (Figure 6A, I–V). For each group, the major GO class represented was identified (Table S3). For example, Groups I, II and IV correspond to genes whose expression strongly correlates with the expression of TTG2 and GL2, and which are enriched in the categories of metabolism, protein synthesis, development and interaction with the environment. In contrast, the expression of SCL8 strongly correlates with Group V, which is primarily enriched in genes involved in cellular transport. These analyses permit us to start narrowing down the specific sets of “trichome genes” that are likely downstream of the second tier regulators (Figure 7), providing a set of candidate genes to continue expanding the regulatory network.

### The Relationship between TTG2 and GL2

The expression of *GL2* and *TTG2* follow a very similar pattern after *GL3* and *GL1* induction (Figure 5). Moreover, the co-expression analyses suggest that the functions of GL2 and TTG2 largely overlap (Figure 6A, B), which is consistent with the similar arrest at the trichome initial stage observed in both mutants [15]. Although *GL2* mRNA levels are not affected in the *ttg2-3* mutant, the expression of a dominant negative version of TTG2 (TTG2-SRDX) almost completely abolished *GL2* expression, suggesting that GL3/GL1, TTG2 and GL2 may form a feed forward loop (Figure S9A and Figure 7), by which TTG2 would control, at least in part, *GL2* expression [22]. To determine whether TTG2 directly controls *GL2*, we performed ChIP experiments on p35S::TTG2-GFP plants, using αGFP. Although we detected *in vivo* binding of TTG2 to its own promoter (Figure S9B), consistent with the proposed positive feedback regulation of *TTG2* [22], we failed to detect *in vivo* binding of TTG2 to *GL2* (Figure S9C). From these results, we conclude that, while the regulatory function of TTG2 and GL2 largely overlap, it is unlikely that TTG2 is directly controlling *GL2* expression. They could be functioning together to modulate the expression of down-stream genes, or TTG2 might indirectly control *GL2* expression.

### Conclusions

Here, we describe the first steps towards establishing the regulatory network involved in the differentiation of epidermal cells into trichomes in *Arabidopsis*. By combining ChIP-chip and genome-wide expression analyses, we have identified direct targets shared by the first tier trichome selectors, GL3 and GL1, in addition to a number of genes putatively controlled by one or the other regulator, most likely as part of regulatory complexes with other characterized R2R3-MYB or bHLH factors, respectively. Among the GL3/GL1 direct targets, at least four transcription factors constitute the second tier regulators of the network hierarchical structure. Co-expression analyses of genes specifically associated with trichome induction were utilized to identify candidate genes downstream of each of the four second-tier regulators, further delineating lower tiers in the network architecture. These studies identified some of the earliest steps involved in trichome initiation, while providing a number of candidate genes that may participate in trichome formation.

## Material and Methods

### Plant Material and Culture

The *Arabidopsis thaliana gl3 egl3* pGL3::GL3-YFP, *gl1* pGL3::GL3-YFP, *gl1* pGL1::GL1-YFP-MYC, *gl3 egl3* pGL3::GL3-GR, *gl1* pGL1::GL1-GR seed stocks have been previously described [12],[13]. Plants were grown on soil containing 100 µM BASTA (Liberty™, AgrEvo) (*gl3 egl3* pGL3::GL3-GR and *gl1* pGL1::GL1-GR) or MS media supplemented with 50 µM kanamycin (*gl3 egl3* pGL3::GL3-YFP, *gl1* pGL1::GL1-YFP-MYC, *gl1* pGL3::GL3-YFP, p35S::TTG2-GFP) at 22°C, under a photoperiod of 16 hours of light and 8 hours dark, unless otherwise indicated.

### RNA Extraction

For DEX treatments experiments, 15 days-old seedlings were transferred from plain MS media to MS media containing 30 µM DEX or 2% ethanol (Mock). DEX was kept as a 3 mM solution in ethanol at −20°C. Green tissues or whole seedlings were collected 4 and 24 hours after DEX treatment and frozen immediately in N_2_(l). Approximately 30 to 40 seedlings were used for each RNA extraction. Plant materials were ground in liquid nitrogen and homogenized in 7.5 ml Trizol reagent. After incubation at room temperature for 5 min, the insoluble material from the homogenate was removed by centrifugation at 12,000×g for 10 min at 4°C, supernatant transferred to a fresh tube and 1.5 ml chloroform was added and mixed by vortexing for 30 sec. Samples were incubated at room temperature for 3 min followed by centrifugation at 10,000×g for 15 min at 4°C. RNA was precipitated from the aqueous phase by mixing the aqueous phase with 3.75 ml isopropyl alcohol. Following incubation at room temperature for 20 min, the samples were centrifuged at 10,000×g for 10 min at 4°C. The RNA precipitates were washed with 10 ml of 70% ethanol and centrifuged again. RNA pellets were dried for 10 min at room temperature and then dissolved in 150 µL nuclease free water by incubating at 60°C for 10 min. RNA samples were further concentrated through Qiagen RNesay® mini columns following the RNeasy mini protocol for RNA cleanup protocol from the manufacturer.

### qRT-PCR Gene Expression Analyses

Approximately 100 mg of green tissues were used for each RNA extraction by using Qiagen RNesay mini columns following the manufacture's instruction. After DNase treatment using RQ1 RNase-free DNase (Promega), reverse transcription (RT) reactions were performed using Superscript II reverse transcriptase (Invitrogen) on approximately 100 ng of total RNA from each sample after DNase treatment (Promega) for 30 min at room temperature. Real-time RT-PCR (qRT-PCR) was performed using iQ SYBR Green Supermix (BIO-RAD) on an iCycler equipment (BioRad). Primers for qRT-PCR were designed to generate 80 bp to 100 bp fragments (See Table S4). We used *At1g13320*, which has been reported to be an appropriate reference gene for qRT-PCR [56], as an internal reference to normalize expression ratios. The qRT-PCR analyses of the test and reference genes were performed simultaneously, following normalization by calculating the fold ratios between test samples and reference gene. Ct values of test samples obtained from qRT-PCR, *Ct_sample_* were subtracted by Ct values of reference, *Ct_ref_*, then the ratios of DEX and Mock were calculated using normalized values using the following equation:
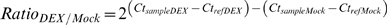



Where 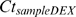 and 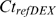 are Ct values of sample and reference genes in DEX treated plants, respectively, and 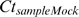 and 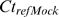 are Ct values of sample and reference genes in Mock treated plants, respectively. Three biological independent materials were used.

### Microarray Genome-Wide Expression Analyses

Two to four biological independent materials were used for RNA preparation. The integrity and concentration of the RNA was verified by capillary electrophoresis using a Bioanalyzer 2100 (Agilent). Sample preparation for hybridization and detection were according to Affymetrix protocols. Raw data (.CEL files) were obtained from the hybridization of *Arabidopsis* Affymetrix ATH1 Arrays with the samples described in Table S2. Whole tissues of *gl1* and wild type, and green tissues of *gl3 egl3* and GL1-GR and GL3-GR plants were used for RNA extraction. Microarray data analyses were performed using the R software with AffylmGUI of the Bioconductor package [57]. The data was normalized by GCRMA prior to further analysis. For the calculation of DEX induced ratios, values from DEX-treated samples were divided by ones from Mock-treated samples, resulting in the DEX/Mock ratios. The ratios of wild type versus mutant (Wild type/mutants ratios) were calculated by wild type expression values divided. Ratios were subjected to Student's *t*-test statistical analyses with a cut-off value of *P*<0.01.

Meta analysis was performed as described [34]. Briefly, *P* values of a gene of each microarray experiment was integrated using Fisher's inverse method:
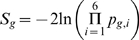
where 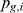 is the *P* value for gene *g* in the experiment *i*. *P* values were integrated from six experiments consisting of 4 and 24 hours DEX inductions of plants carrying pGL1::GL1-GR or pGL3::GL3-GR genes, and wild type and *gl1* or *gl3 egl3* mutants. 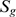 corresponds to the chi-square distribution with 12 degrees of freedom. Then, the *P* value for gene *g* based on the integral analysis of all the datasets was calculated using the 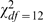 distribution. For controlling the False Discovery Rate (FDR), *q*-values were calculated by the R module, QVALUE [58] and genes that showed *q* value less than 0.05 were considered for further analyses. The *q*-value of this test measures the minimum FDR that is incurred when calling that test significant, whereas the *P* value of a test measures the minimum false positive rate that is incurred when calling that test significant. Using *q*-values, it is possible to assign a measure of significance to each one of many tests.

### ChIP and ChIP-chip Experiments

Whole seedlings from three-week-old plants grown on soil were subjected to ChIP experiments, which were performed as described [13],[59]. For ChIP-chip experiments, precipitated and input DNA were amplified using the GenomePlex Whole Genome Amplification Kit (Sigma), following the method modified for ChIP-chip [60]. DNA fragmentation, labeling, hybridization, washes and detection were performed following the Affymetrix 100K protocol (http://www.affymetrix.com/products/arrays/specific/100k.affx). CEL files were further analyzed by MAT (Model-based Analysis of Tiling array; http://chip.dfci.harvard.edu/˜wli/MAT/) [23] using the following parameters: BandWith = 300, MaxGap = 300, MinProbe = 10 and Pvalue = 0.001. Peaks consisting of continuous probes with significant MAT scores were evaluated using IGB (Integrated Genome Browser, Affymetrix) with the additional criteria that the minimum gap should be less than 100 bp. We defined target genes as those for which 3 kbp upstream regions contained at least one peak showing significant MAT scores.

### Computational Analysis of the ChIP-chip Data

To investigate the distribution of binding sites, relative MAT score were calculated. Each MAT score for GL1 and GL3 was divided by the average MAT scores of the corresponding negative controls obtained from ChIP-chip experiments with IgG on the pGL3::GL3-YFP or pGL1::GL1-YFP-MYC plants, or on wild type plants, which do not carry GFP, with αGFP. We first aligned the transcription start site (TSS) of all *Arabidopsis* genes and divided the genomic regions into 50 bins (60 bp each) in the [−3,000; +3,000] interval, followed by plotting means of relative MAT scores based on the bins. Heatmaps of expression profiles were drawn with TM4 (TIGR, http://www.tm4.org/) [61]. Hierarchical clustering with metrics of Euclidean distances and average linkage clustering was utilized for making heatmaps. We used custom-made Perl scripts (available at http://grassius.org/help.html).

### Pearson's Correlation Coefficient (PCC) Analyses

PCC of GL2, TTG2, SCL8 and MYC1 with each of approximately 500 genes affected by GL1 and/or GL3 were obtained from ATTED-II (http://www.atted.bio.titech.ac.jp/) [62]. Five clusters were chosen manually after hierarchical clustering of PCC distribution of *GL2*, *TTG2*, *SCL8* and *MYC1* with genes affected by GL1 and GL3. Main GO distributions for each class of genes were determined using the FunCat application ([63]; http://mips.gsf.de/proj/funcatDB/search_main_frame.html) from data in MIPS.

### Microarray Data accession Numbers

All the microarray data generated as part of this study has been deposited in the Gene Expression Omnibus (http://www.ncbi.nlm.nih.gov/geo/) with accession numbers GSE12551, GSE12522 and GSE13090.

## Supporting Information

Figure S1 Summary of ChIP-chip results. (A) Representative entire signal distributions of the five *Arabidopsis* chromosomes from GL1 (brown) and GL3 (blue) ChIP-chip analyses. (B–C) Representative IGB results corresponding to (B) GL1 and (C) GL3, showing the genomic regions containing significant enriched signals. The y-axis indicates MAT score. The gene annotation, shown in green, was obtained from TAIR. Large boxes correspond to exons; small boxes to untranslated regions and lines to introns. Gene orientations are indicated on the left side of the picture. Arrow-heads represent *cis*-element that have been experimentally demonstrated as important for gene expression. (D) Venn diagrams summarize the ChIP-chip results for GL1 and GL3.
(1.5 MB TIF)

Figure S2 Distributions of signals in the GL1 and GL3 ChIP-chip experiments. (A) Distribution of GL1 (middle) and GL3 (right) binding regions relative to the overall *Arabidopsis* genome gene structure (left). For this analysis, the components of the genome were divided into intergenic (light blue), 5′UTR (red), 3′UTR (yellow), intron (green) and CDS (dark blue) segments, as shown on the right of the graphs. (B) Distribution of relative MAT ChIP-chip mean scores for GL1 (red) and GL3 (blue) on 50 bins (60 bp each) corresponding to the [−3,000; +3,000] region flanking the TSS.
(0.2 MB TIF)

Figure S3 Validation of GL1 ChIP-chip results. (A) A set of 15 random genes showing significant MAT scores in the GL1 ChIP-chip experiments was selected for validation by conventional ChIP-PCR. The different regions are indicated by the corresponding peak positions in the *Arabidopsis* genome, and the corresponding IGB image of the region is displayed. Black squares indicate the position of the fragments amplified by PCR. The PCR validation includes the corresponding input control (Input), the IgG negative control (IgG) and the precipitated fraction by αGFP, as indicated above the picture. The IGB presentation of the region chosen for standard ChIP-PCR is shown on the left. (B) A set of 20 random genes showing significant MAT scores in the ChIP-chip experiments with GL3 was selected for validation by conventional ChIP-PCR. The different regions are indicated by the corresponding peak positions in the *Arabidopsis* genome, and the corresponding IGB image of the region is displayed. Black squares indicate the position of the fragments amplified by PCR. The PCR validation includes the corresponding input control (Input), the IgG negative control (IgG) and the precipitated fraction by αGFP, as indicated above the picture. The IGB presentation of the region chosen for standard ChIP-PCR is shown on the left.
(3.1 MB TIF)

Figure S4 Characteristics of the new GL3/GL1 direct targets. (A) Diagrammatic representation of the protein structures of six “unknown genes” based on the presence of domains identified from TAIR or by PHYRE. (B–D) Genevestigator (https://www.genevestigator.ethz.ch/gv/index.jsp) analyses of these genes in (B) different tissues, (C) developmental stages, and (D) under various conditions.
(1.7 MB TIF)

Figure S5 Identification of GL1/GL3 direct targets from genes affecting trichome development. Representative ChIP experiments performed with αGFP or IgG (negative control) on two biological replicates (#1 and #2) on *gl3 egl3* pGL3::GL3-YFP, *gl1* pGL1::GL1-YFP-MYC or *gl1* pGL3::GL3-YFP plants. A gene was concluded to be a GL1 or GL3 direct target only when no signal was detected in the IgG control, and duplicates gave the same results.
(3.1 MB TIF)

Figure S6 Comparison of differentially expressed genes at different time points after the induction of GL1-GR and GL3-GR with DEX. (A) Venn diagrams comparing alterations in mRNA accumulation after 4 hours or 24 hours of DEX induction of pGL1::GL1-GR (GL1, left) or pGL3::GL3-GR (GL3, right). (B) Venn diagrams comparing the overlap of differentially expressed at different time points after the induction of GL1 and GL3, with the identified direct target genes for each of these two regulators shown.
(0.3 MB TIF)

Figure S7Functional classification of the 513 genes comprising the minimal set of “Trichome genes”. Genes were divided into five groups based on the cluster analysis of PCC (Figure 6). The probability *p*, calculated based on statistics of hyper geometric distribution, was converted to 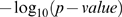 for clarity. In this graph, 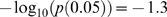.(0.3 MB TIF)

Figure S8 Structure of the genomic region corresponding to *At3g50790/At3g50800* and corresponding IGB representation of the GL3 and GL1 enriched sites.
(0.8 MB TIF)

Figure S9 Regulatory relationships between GL3, TTG2 and GL1. (A) Regulatory motif showing that GL3/GL1 directly control *TTG2* and *GL2* expression, and also that TTG2 is involved in its own regulation. (B) ChIP experiments on *gl3 egl3* pGL3::GL3-YFP, *gl1* pGL1::GL1-YFP-MYC or *gl1* pGL3::GL3-YFP plants demonstrate that GL3 and GL1 bind *in vivo* the *TTG2* promoter, and that GL3 binding requires *GL1*. (C) ChIP experiments in p35S::TTG2-GFP plants demonstrate that TTG2 binds its own promoter, but fails to recognize the promoter region of *GL2*.
(0.6 MB TIF)

Table S1 List of genes participating in trichome formation and summary of ChIP-chip and standard ChIP experiments.
(0.05 MB XLS)

Table S2 Gene list of GL1 and/or GL3 regulated genes from meta-analysis (*q*<0.05).
(0.02 MB XLS)

Table S3 PCC scores of GL1 and GL3 controlled for co-expression with *GL2*, *TTG2*, *SCL8* and *MYC1*.
(0.05 MB XLS)

Table S4 List of primers used in this study.
(0.04 MB XLS)
