# Supplementary figures and images for: A Systems Approach Reveals Regulatory Circuitry for Arabidopsis Trichome Initiation by the GL3 and GL1 Selectors
Source: PLoS Genet. 2009 Feb 27;5(2):e1000396. doi: 10.1371/journal.pgen.1000396 (PMC2642726; doi:10.1371/journal.pgen.1000396)

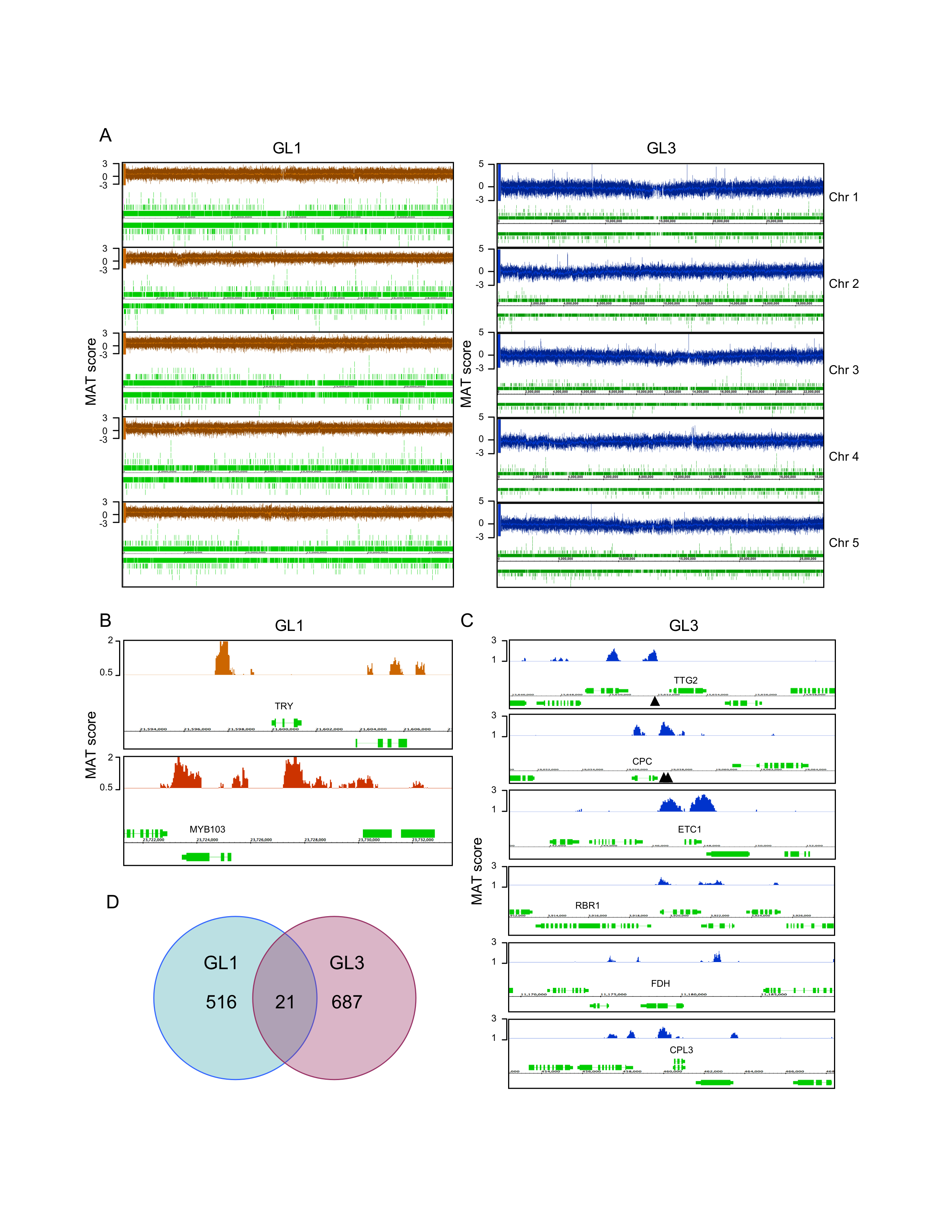

Supplement: Figure S1 — Summary of ChIP-chip results. (A) Representative entire signal distributions of the five Arabidopsis chromosomes from GL1 (brown) and GL3 (blue) ChIP-chip analyses. (B–C) Representative IGB results corresponding to (B) GL1 and (C) GL3, showing the genomic regions containing significant enriched signals. The y-axis indicates MAT score. The gene annotation, shown in green, was obtained from TAIR. Large boxes correspond to exons; small boxes to untranslated regions and lines to introns. Gene orientations are indicated on the left side of the picture. Arrow-heads represent cis-element that have been experimentally demonstrated as important for gene expression. (D) Venn diagrams summarize the ChIP-chip results for GL1 and GL3. (1.5 MB TIF) [file pgen.1000396.s001.tif]

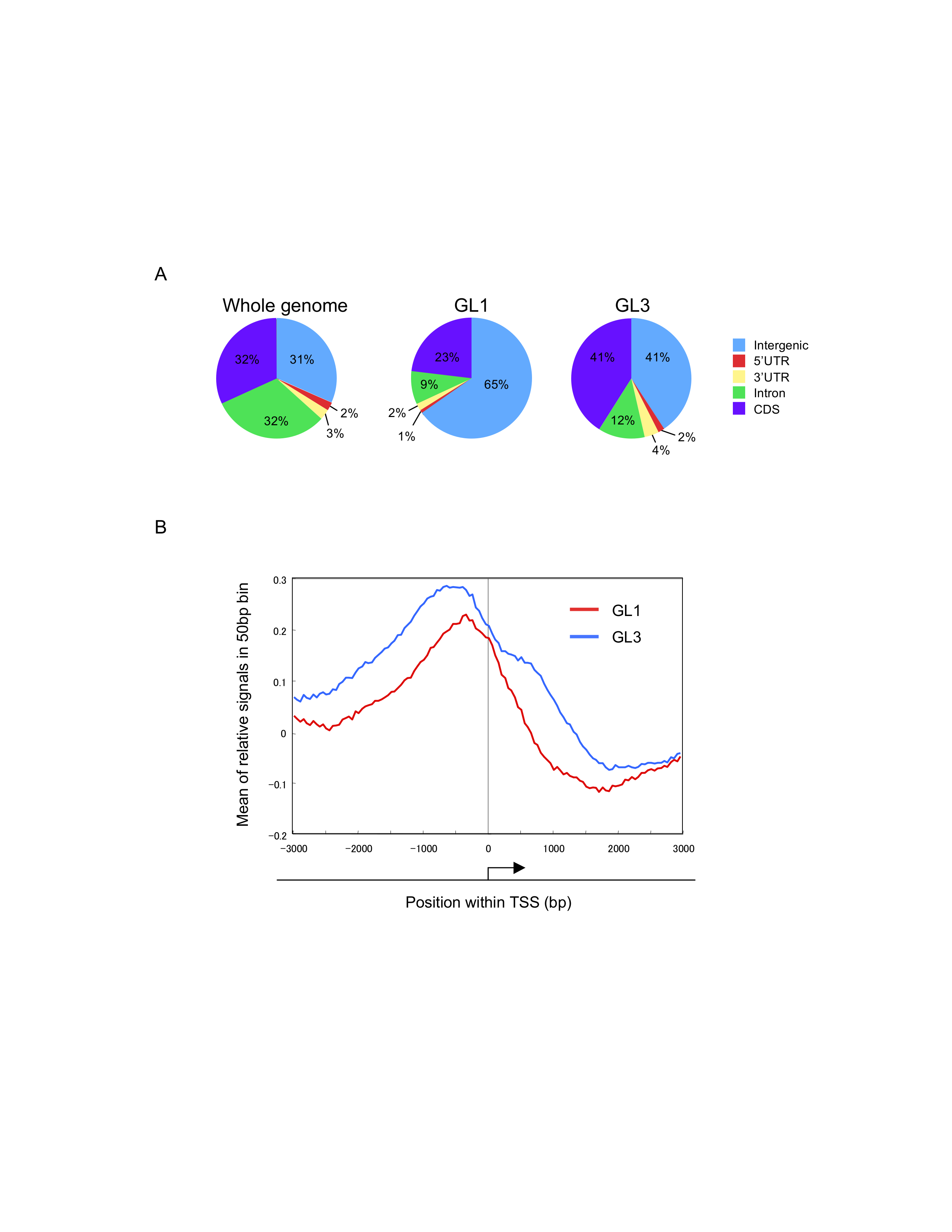

Supplement: Figure S2 — Distributions of signals in the GL1 and GL3 ChIP-chip experiments. (A) Distribution of GL1 (middle) and GL3 (right) binding regions relative to the overall Arabidopsis genome gene structure (left). For this analysis, the components of the genome were divided into intergenic (light blue), 5′UTR (red), 3′UTR (yellow), intron (green) and CDS (dark blue) segments, as shown on the right of the graphs. (B) Distribution of relative MAT ChIP-chip mean scores for GL1 (red) and GL3 (blue) on 50 bins (60 bp each) corresponding to the [−3,000; +3,000] region flanking the TSS. (0.2 MB TIF) [file pgen.1000396.s002.tif]

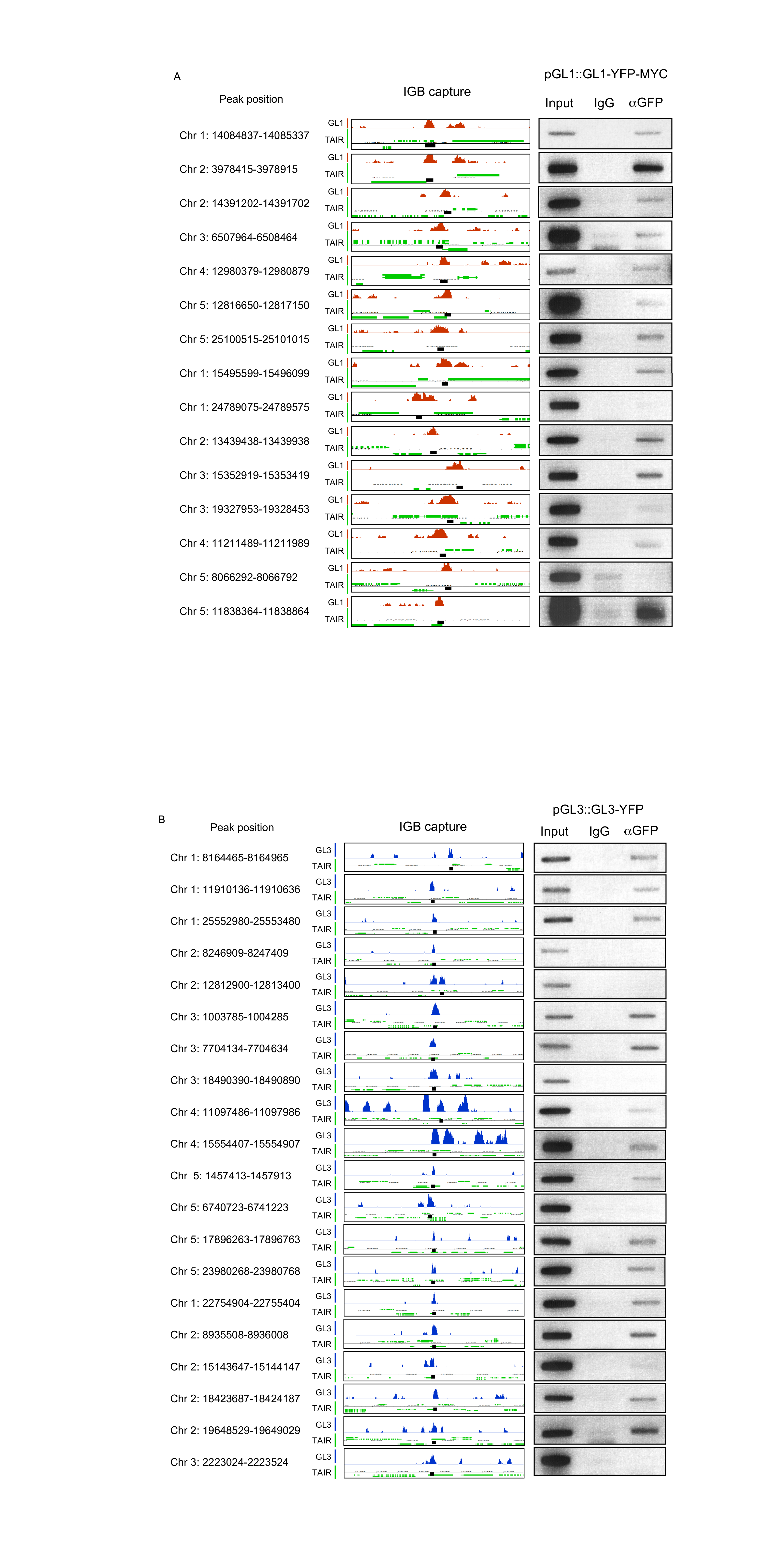

Supplement: Figure S3 — Validation of GL1 ChIP-chip results. (A) A set of 15 random genes showing significant MAT scores in the GL1 ChIP-chip experiments was selected for validation by conventional ChIP-PCR. The different regions are indicated by the corresponding peak positions in the Arabidopsis genome, and the corresponding IGB image of the region is displayed. Black squares indicate the position of the fragments amplified by PCR. The PCR validation includes the corresponding input control (Input), the IgG negative control (IgG) and the precipitated fraction by αGFP, as indicated above the picture. The IGB presentation of the region chosen for standard ChIP-PCR is shown on the left. (B) A set of 20 random genes showing significant MAT scores in the ChIP-chip experiments with GL3 was selected for validation by conventional ChIP-PCR. The different regions are indicated by the corresponding peak positions in the Arabidopsis genome, and the corresponding IGB image of the region is displayed. Black squares indicate the position of the fragments amplified by PCR. The PCR validation includes the corresponding input control (Input), the IgG negative control (IgG) and the precipitated fraction by αGFP, as indicated above the picture. The IGB presentation of the region chosen for standard ChIP-PCR is shown on the left. (3.1 MB TIF) [file pgen.1000396.s003.tif]

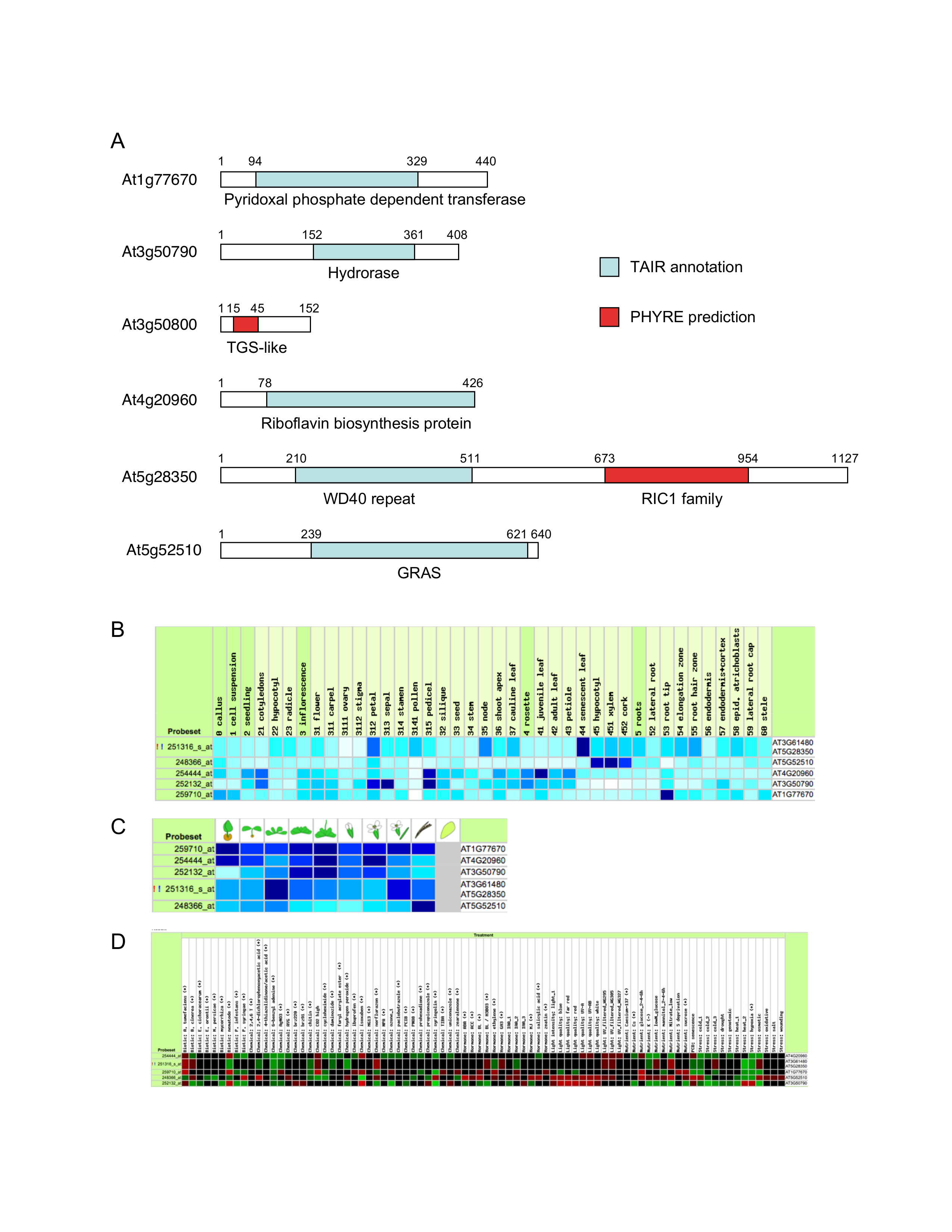

Supplement: Figure S4 — Characteristics of the new GL3/GL1 direct targets. (A) Diagrammatic representation of the protein structures of six “unknown genes” based on the presence of domains identified from TAIR or by PHYRE. (B–D) Genevestigator (https://www.genevestigator.ethz.ch/gv/index.jsp) analyses of these genes in (B) different tissues, (C) developmental stages, and (D) under various conditions. (1.7 MB TIF) [file pgen.1000396.s004.tif]

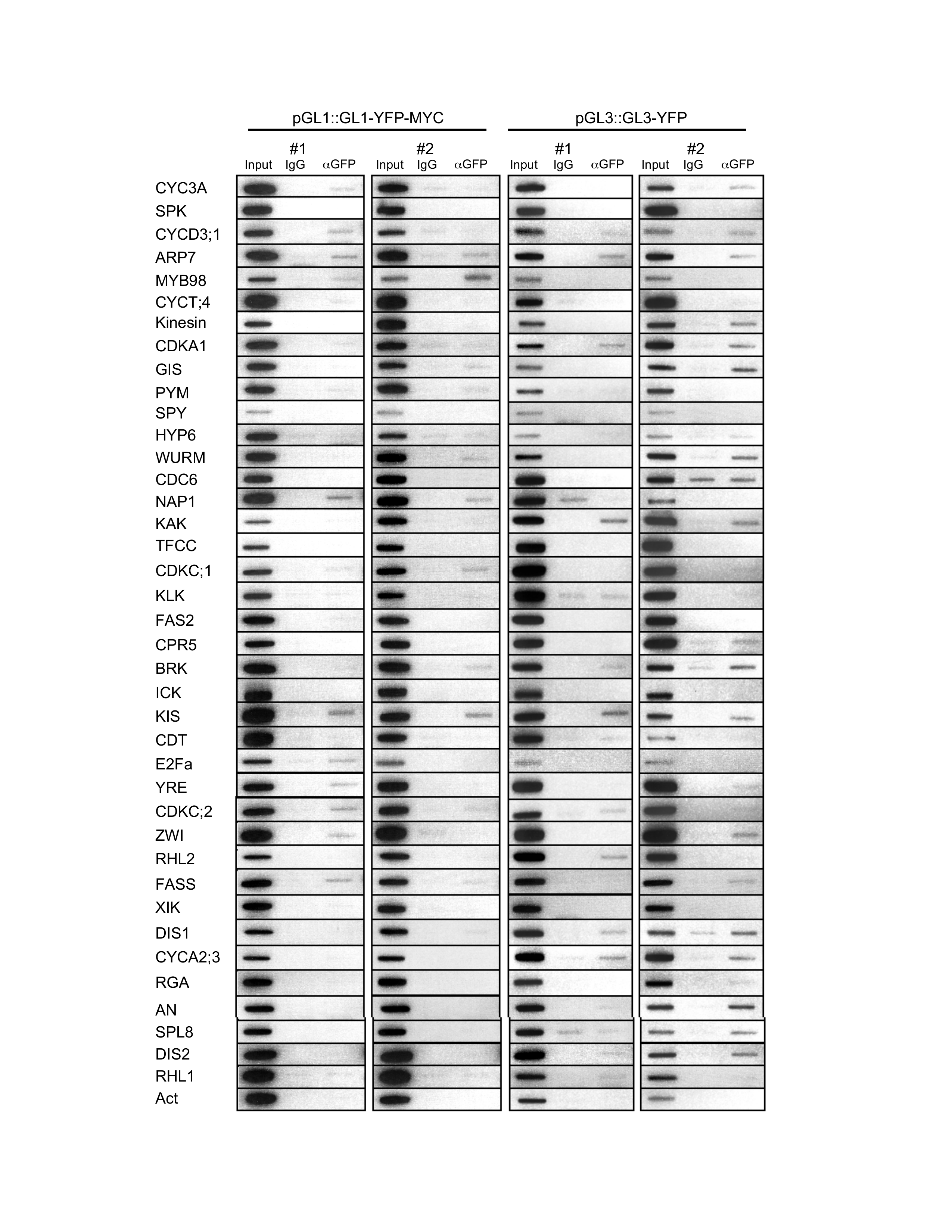

Supplement: Figure S5 — Identification of GL1/GL3 direct targets from genes affecting trichome development. Representative ChIP experiments performed with αGFP or IgG (negative control) on two biological replicates (#1 and #2) on gl3 egl3 pGL3::GL3-YFP, gl1 pGL1::GL1-YFP-MYC or gl1 pGL3::GL3-YFP plants. A gene was concluded to be a GL1 or GL3 direct target only when no signal was detected in the IgG control, and duplicates gave the same results. (3.1 MB TIF) [file pgen.1000396.s005.tif]

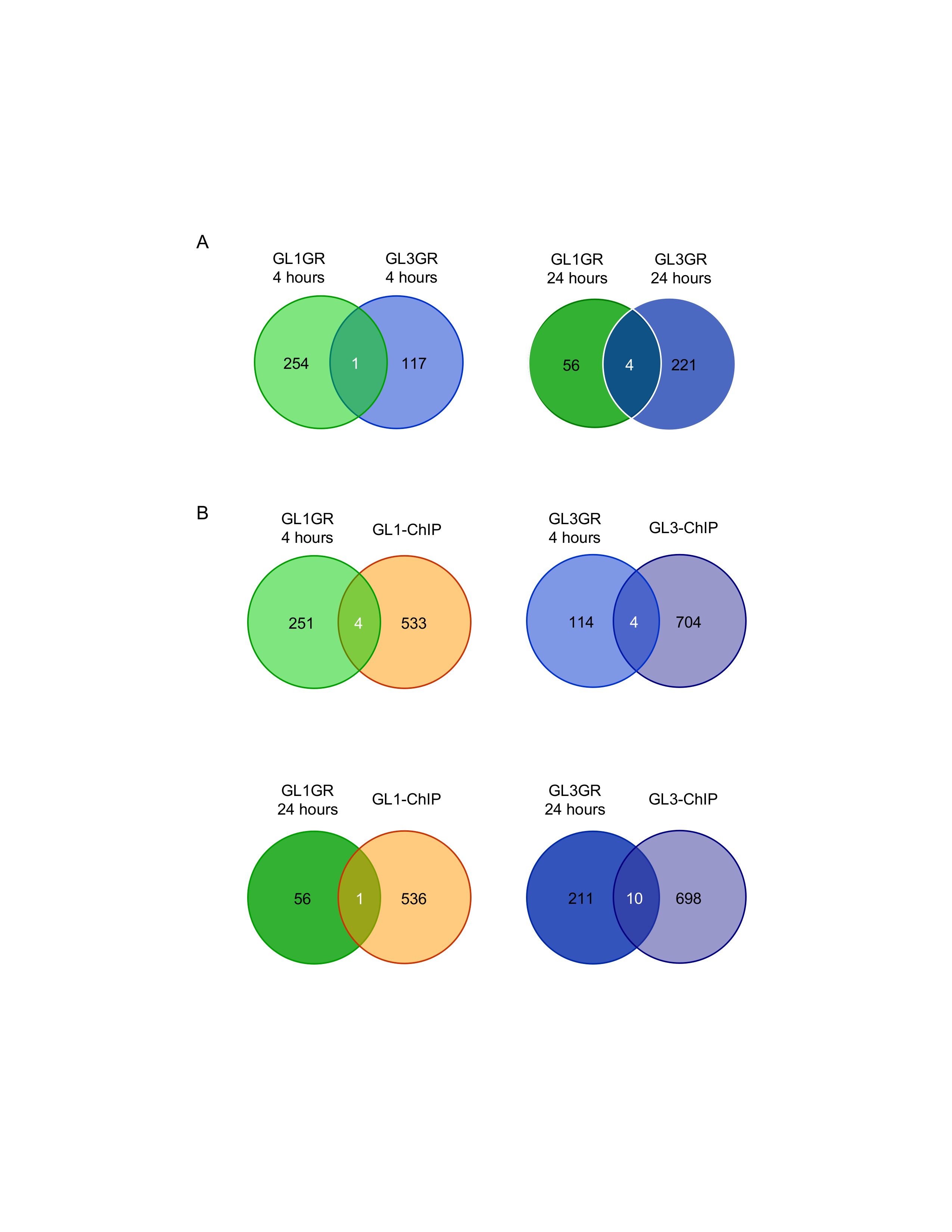

Supplement: Figure S6 — Comparison of differentially expressed genes at different time points after the induction of GL1-GR and GL3-GR with DEX. (A) Venn diagrams comparing alterations in mRNA accumulation after 4 hours or 24 hours of DEX induction of pGL1::GL1-GR (GL1, left) or pGL3::GL3-GR (GL3, right). (B) Venn diagrams comparing the overlap of differentially expressed at different time points after the induction of GL1 and GL3, with the identified direct target genes for each of these two regulators shown. (0.3 MB TIF) [file pgen.1000396.s006.tif]

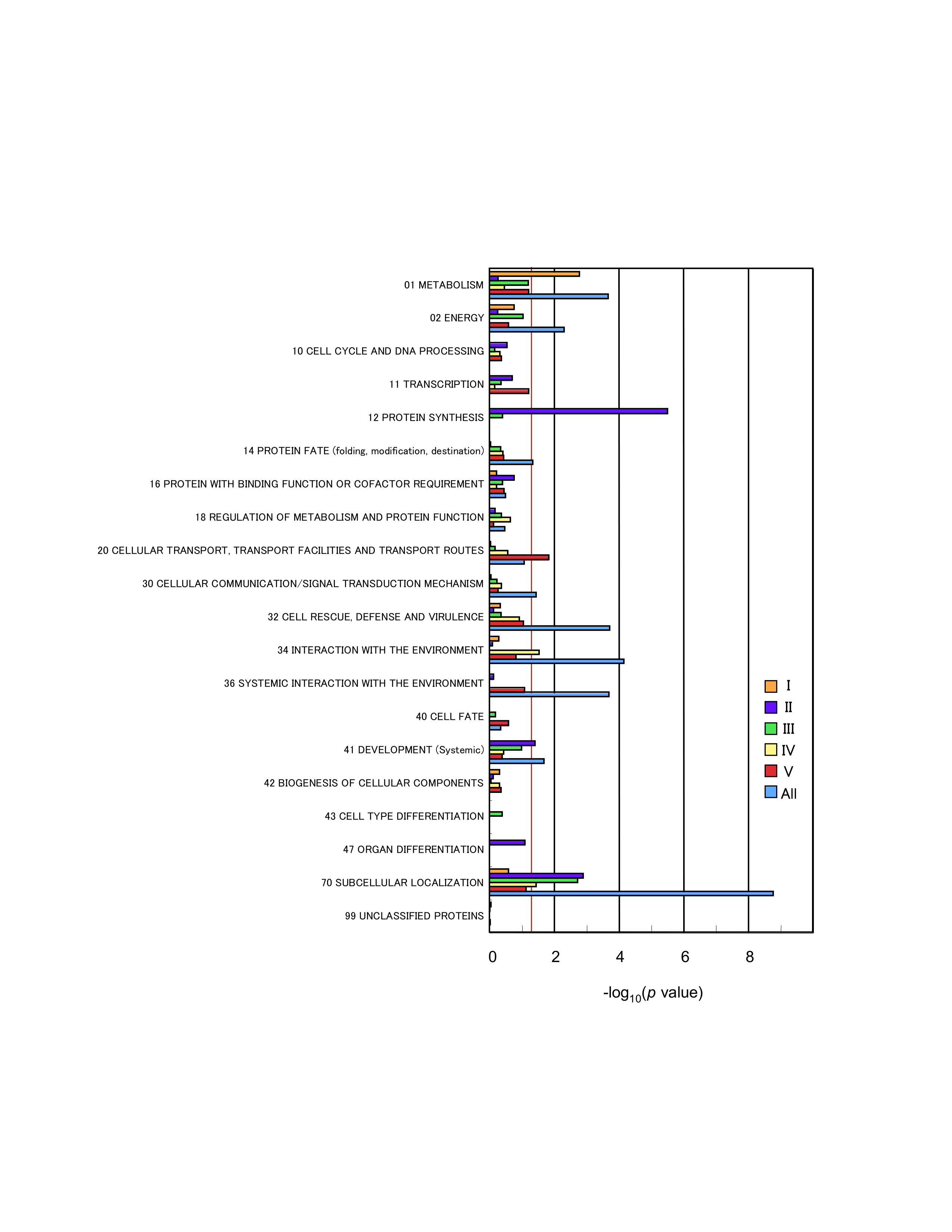

Supplement: Figure S7 — Functional classification of the 513 genes comprising the minimal set of “Trichome genes”. Genes were divided into five groups based on the cluster analysis of PCC (Figure 6). The probability p, calculated based on statistics of hyper geometric distribution, was converted to for clarity. In this graph, . (0.3 MB TIF) [file pgen.1000396.s007.tif]

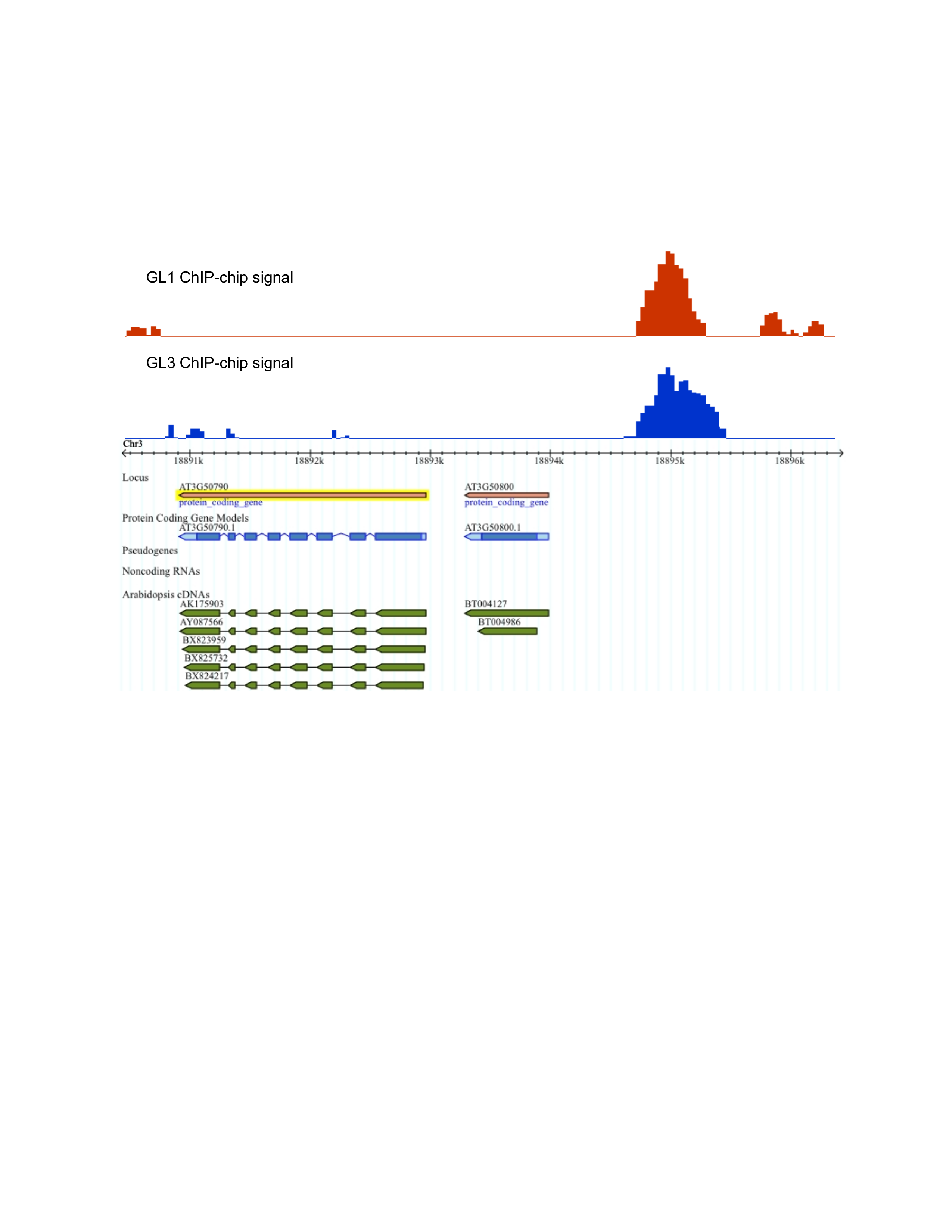

Supplement: Figure S8 — Structure of the genomic region corresponding to At3g50790/At3g50800 and corresponding IGB representation of the GL3 and GL1 enriched sites. (0.8 MB TIF) [file pgen.1000396.s008.tif]

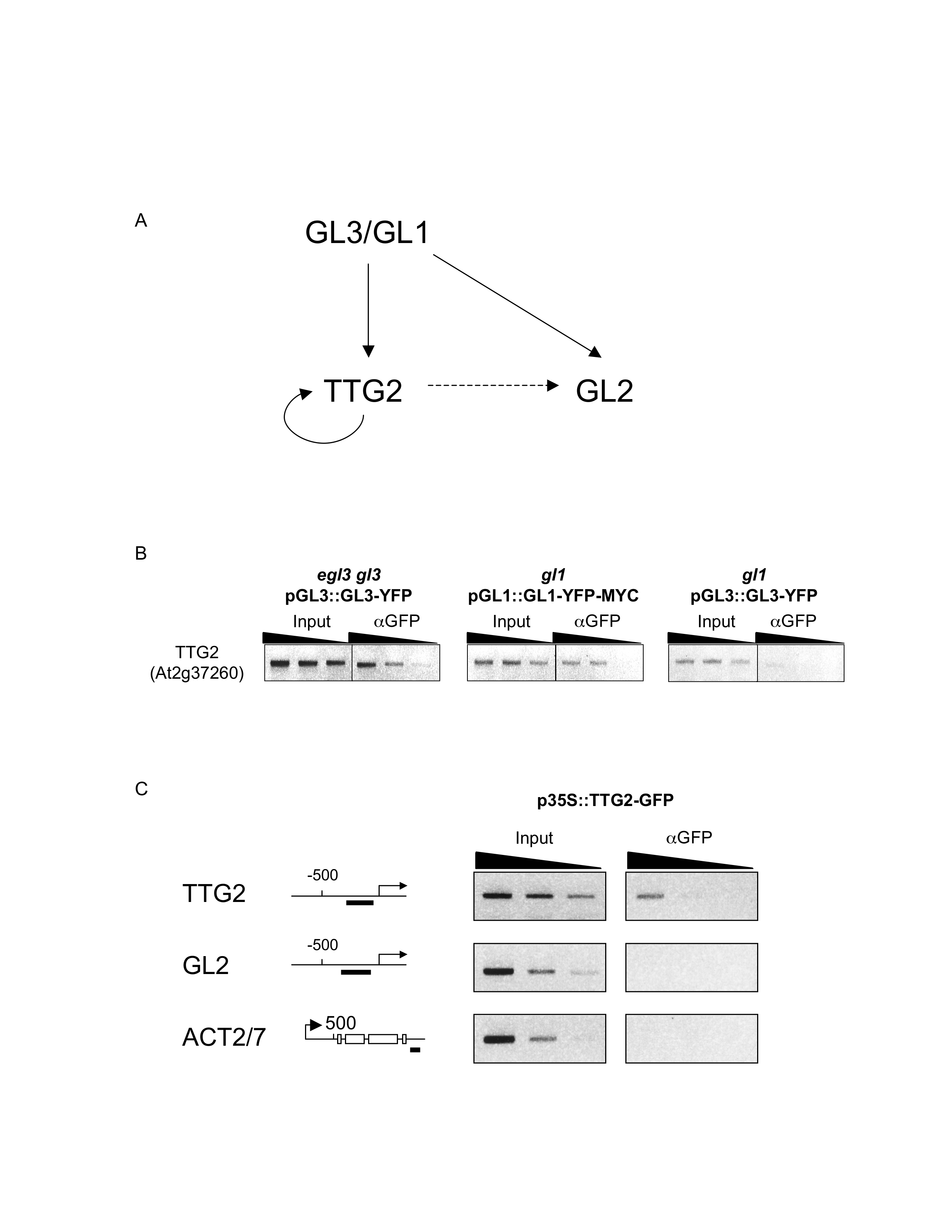

Supplement: Figure S9 — Regulatory relationships between GL3, TTG2 and GL1. (A) Regulatory motif showing that GL3/GL1 directly control TTG2 and GL2 expression, and also that TTG2 is involved in its own regulation. (B) ChIP experiments on gl3 egl3 pGL3::GL3-YFP, gl1 pGL1::GL1-YFP-MYC or gl1 pGL3::GL3-YFP plants demonstrate that GL3 and GL1 bind in vivo the TTG2 promoter, and that GL3 binding requires GL1. (C) ChIP experiments in p35S::TTG2-GFP plants demonstrate that TTG2 binds its own promoter, but fails to recognize the promoter region of GL2. (0.6 MB TIF) [file pgen.1000396.s009.tif]
